# Validity of Dietary Assessment in Athletes: A Systematic Review

**DOI:** 10.3390/nu9121313

**Published:** 2017-12-02

**Authors:** Louise Capling, Kathryn L. Beck, Janelle A. Gifford, Gary Slater, Victoria M. Flood, Helen O’Connor

**Affiliations:** 1 Discipline of Exercise and Sport Science, Faculty of Health Sciences, The University of Sydney, Lidcombe, NSW 2141, Australia; janelle.gifford@sydney.edu.au (J.A.G.); vicki.flood@sydney.edu.au (V.M.F.); helen.oconnor@sydney.edu.au (H.O.); 2 Sport Performance Innovation and Knowledge Excellence, Queensland Academy of Sport, Brisbane, QLD 4111, Australia; 3 School of Sport Exercise and Nutrition, College of Health, Massey University, Auckland 0745, New Zealand; k.l.beck@massey.ac.nz; 4 School of Health and Sport Sciences, University of the Sunshine Coast, Maroochydore, QLD 4558, Australia; gslater@usc.edu.au; 5 Charles Perkins Centre, The University of Sydney, Camperdown, NSW 2006, Australia; 6 Western Sydney Local Health District, Westmead, NSW 2145, Australia

**Keywords:** dietary assessment, food record, FFQ, biomarker, doubly labeled water, energy intake, validity, athletes, sports nutrition

## Abstract

Dietary assessment methods that are recognized as appropriate for the general population are usually applied in a similar manner to athletes, despite the knowledge that sport-specific factors can complicate assessment and impact accuracy in unique ways. As dietary assessment methods are used extensively within the field of sports nutrition, there is concern the validity of methodologies have not undergone more rigorous evaluation in this unique population sub-group. The purpose of this systematic review was to compare two or more methods of dietary assessment, including dietary intake measured against biomarkers or reference measures of energy expenditure, in athletes. Six electronic databases were searched for English-language, full-text articles published from January 1980 until June 2016. The search strategy combined the following keywords: diet, nutrition assessment, athlete, and validity; where the following outcomes are reported but not limited to: energy intake, macro and/or micronutrient intake, food intake, nutritional adequacy, diet quality, or nutritional status. Meta-analysis was performed on studies with sufficient methodological similarity, with between-group standardized mean differences (or effect size) and 95% confidence intervals (CI) being calculated. Of the 1624 studies identified, 18 were eligible for inclusion. Studies comparing self-reported energy intake (EI) to energy expenditure assessed via doubly labelled water were grouped for comparison (*n* = 11) and demonstrated mean EI was under-estimated by 19% (−2793 ± 1134 kJ/day). Meta-analysis revealed a large pooled effect size of −1.006 (95% CI: −1.3 to −0.7; *p* < 0.001). The remaining studies (*n* = 7) compared a new dietary tool or instrument to a reference method(s) (e.g., food record, 24-h dietary recall, biomarker) as part of a validation study. This systematic review revealed there are limited robust studies evaluating dietary assessment methods in athletes. Existing literature demonstrates the substantial variability between methods, with under- and misreporting of intake being frequently observed. There is a clear need for careful validation of dietary assessment methods, including emerging technical innovations, among athlete populations.

## 1. Introduction

Adequate dietary intake is important for normal growth and development, maintaining health and well-being, reducing the risk of illness and injury, and optimizing sports performance [1]. Individual dietary requirements are influenced by a range of factors such as age, gender, body mass, stature, and growth and development needs (for child or adolescent athletes) [1,2]. In addition, a range of sport-specific factors, such as type of sport, training volume and intensity also influence dietary requirements, which are not static due to the periodisation of training load across days, weeks, or months of a competitive season [1,2]. Athletes have special nutrition needs that usually encompass a higher energy requirement to account for greater energy expenditure, increased protein and carbohydrate requirements to support lean mass accrual and/or maintenance, and glycogen stores, respectively, as well as an increased requirement for certain micronutrients (e.g., iron, calcium, sodium) [1,2,3]. 

Dietary assessment is routinely undertaken by nutrition professionals to evaluate whether an individual is achieving specific health and/or sports nutrition targets [4]. However, accurate dietary assessment of athletes is complex due to the influence of sport-specific factors, such as periodised training, large portion sizes, and the widespread use of rapidly evolving sports foods and supplements. Dietary assessment can be expensive (especially for large numbers of athletes or teams), time consuming and unfortunately due to these constraints, undertaken less frequently. As a result, athletes with inadequate or poor dietary intake may not be readily identified. For example, chronic low energy availability may lead to the modification of body composition, which can compromise health and performance [1,2,3].

Numerous studies have reported on dietary intake of athletes from a wide range of sports [5,6,7,8,9]; with many comparing self-reported intake to calculated energy requirements based on general recommendations and/or sports nutrition guidelines [10,11,12,13,14]. However, the discrepancy between self-reported intake and estimated energy expenditure has been reported in a number of studies involving male and female athletes, across a range of age-groups and a variety of sports [15,16,17]; with substantial differences (11–44%) being observed [18,19].

Dietary assessment methodology recognised as suitable for non-athletes are usually applied in a similar manner to athletes, although specific factors, such as training status, competition level, and nutrition-associated beliefs and dietary practices could influence aspects of the dietary assessment process [19]. Many retrospective methods that rely on self-reporting of intake (e.g., food record, 24-h dietary recall, diet history) are susceptible to measurement error including conscious or sub-conscious exclusion of foods consumed [4,19,20,21]; and, possible change in usual food intake or dietary patterns due to the nature of the dietary intervention itself [22].

While there is currently no gold standard for measuring energy intake, by far the most common dietary assessment method that is applied in sports nutrition research and practice is the food record (FR); where all food and drinks that are consumed are recorded by the participant for a specified number of days (i.e., 3–7 days) [4]. However, it has been widely reported that self-reported FR under-estimates intake, as noted in a number of population groups, including adolescents and children [23], obese individuals [24], and athletes [18,25,26]. Despite the FR being regarded a ‘gold standard’ in dietary assessment, it places a substantial burden on participants to document information truthfully and accurately, as well as a reliance on investigators to code the data correctly using appropriate databases [19,27,28,29]. Additional challenges that are facing an athlete cohort include an increased burden from recording large food intakes, frequent eating occasions, potentially irregular meal patterns, difficulty estimating large portion sizes, and contribution of sports foods and supplements [19].

Recent technological developments involving the use of food photography or electronic images, wearable cameras, and various on-line tools and applications have been shown to improve participant compliance by reducing the burden of recording and enhance the accuracy of data recorded [30,31,32,33]. There is a need for high quality research to assist in identifying dietary methodologies, including emerging technologies, which are valid, as well as feasible, for use in this unique population sub-group.

Despite a clear need for the careful validation of dietary assessment methods in athletes, there has been relatively little attention directed to the applicability and validity of dietary assessment methods in this population sub-group. The purpose of this systematic review was to evaluate studies comparing nutrition intake from two or more dietary assessment methods, including intake measured against biomarkers or reference measures of energy expenditure, in athletes.

## 2. Methods 

### 2.1. Search Strategy 

A systematic literature search was conducted of articles that were published between January 1980 to June 2016 to identify studies comparing the nutrition intake assessed by two or more methods of dietary assessment, including a measure of intake against dietary biomarkers or reference measures of energy expenditure, in an athlete cohort. Databases searched were AUSPORT MED (via Informit Online), CINAHL (via EBSCO), EMBASE (via EBSCO), MEDLINE (via OvidSP), SPORTDiscus (via EBSCO) and the Web of Science. Ongoing electronic monitoring was established to assess the inclusion of eligible recent publications.

The search strategy combined the following keywords: diet (e.g., energy intake, nutritional status, nutritional requirement, food group); nutrition assessment (e.g., food frequency questionnaire, food record, diet score, diet survey, energy expenditure, biomarkers); athlete (e.g., elite athlete, team sport, collegiate, Olympic athlete); and, validity (e.g., under-reporting, measurement error). In addition, the reference lists of all the retrieved papers and relevant reviews were manually searched for eligible papers. Following the search, we completed a PRISMA (Preferred Reporting Items for Systematic Reviews and Meta-Analyses) [34] informed systematic review process.

### 2.2. Selection of Studies

Study participants had to be ‘athletes’ (i.e., amateur, collegiate, tertiary, professional, national, or international calibre), as defined by the study authors. Included studies needed to validate at least one dietary assessment method (e.g., food frequency questionnaire, 24-h dietary recall, food record) against at least one other dietary assessment method or a biological (blood or urine) measure for nutrient status (e.g., 24-h nitrogen excretion, antioxidant concentrations). In addition, studies that compared reported energy intake to measured energy expenditure (e.g., doubly labelled water technique, activity records) were also included. Descriptive, cross-sectional, or longitudinal study designs were eligible for inclusion. Studies including participants with a mean age lower than 16 years of age, papers published in a language other than English, case studies, reviews, abstracts, and theses were excluded (Supplementary Material 1). After eliminating duplicates, one reviewer (LC) screened the search results against the eligibility criteria. References that could not be eliminated by title and abstract were retrieved in full and subsequently assessed against the eligibility criteria in duplicate (KB, GS) prior to inclusion.

### 2.3. Data Extraction and Conversions

Data relating to the manuscript, namely author(s), date of publication, and country where the study was conducted were extracted. The institution country of the first author was used if the study country was not described in the paper. Data extracted from each publication included: participant characteristics (i.e., age, sex, sport, and athlete calibre), participant anthropometric (i.e., stature, body mass), or body composition parameters (e.g., percent fat), and dietary assessment methodology (e.g., food record, food frequency questionnaire, 24-h dietary recall). In addition, information on reported energy and macronutrient intake, resting energy expenditure, total energy expenditure, and key statistical comparisons between dietary assessment methods and correlation coefficients, were extracted in conjunction with the main study findings. Where a publication contained insufficient information, attempts were made to contact the study author(s) to obtain the missing details. To aid in the comparison between the included papers, anthropometric parameters reported in imperial units (e.g., feet, inches, pounds) were converted to metric units (e.g., kg, cm) (1 kg = 2.2 pounds; 1 cm = 0.3937 inches). Data reported in calories (e.g., energy intake, energy expenditure) were converted to kilojoules (1 Cal = 4.184 kJ). Where reported, macronutrient values were converted from grams or grams per kilogram body weight to per cent of mean energy intake using Atwater factors (i.e., protein 17 kJ/g or 4 Cal/g; fat 37 kJ/g or 9 Cal/g; carbohydrate 16 kJ/g or 4 Cal/g) [35]. Extracted data were presented as mean and standard deviation (SD) where reported. Weighted means were calculated for age, anthropometric variables and the differences between reported energy intake and measured energy expenditure.

### 2.4. Assessment of Methodological Quality 

The methodological quality of all the included studies were independently assessed by three researchers (LC reviewed all of the papers; KB and JAG shared the role of second reviewer on the same studies) using a modified assessment scale that was created by Downs and Black [36]. Using the scale, 18 of the 27 criteria that logically applied to the study designs were retained, while items 4, 8, 9, 13, 14, 17, 21, 23, 24 were deemed not relevant to the included studies and were excluded. Three additional items from a nutrition-specific quality criteria checklist were incorporated [37]; specifically, item 8.6 “Was clinical significance as well as statistical significance reported?”; item 9 “Are conclusions supported by results with biases and limitations taken into consideration?”; and item 10 “Is bias due to study’s funding or sponsorship unlikely?” Where relevant, other items from the Academy of Nutrition and Dietetics quality criteria checklist [37] (i.e., items 2.1, 2.3, 3.4, 5.2, 7.2, 7.3, 7.4, 7.5, and 7.6) were considered concurrently to the Downs and Black [36] criteria to provide reviewer clarification when assessing quality ratings. Two variables from a third checklist described by Serra-Majem and colleagues [38] were applied specifically to validation studies to evaluate the validity of the dietary methodology used and the suitability of the method for an athlete cohort. The specific variables that were applied to assess the quality of the validation study were statistics to assess validity (i.e., to support Downs and Black [36] item 18 “Were the statistical tests used to assess the main outcomes appropriate?”); and to assess whether the sample size was adequate to assess validity (i.e., to support Downs and Black [36] item 27 “Did the study have sufficient power to detect a clinically significant effect where the probability value for a difference being due to chance is less than 5%?”) (Supplementary Material 2). Each reviewer checked for internal (intra-rater) validity across items for each included publication. Disagreements in ratings were resolved by discussion and through adjudication with two separate researchers (HO, VMF). No further sub-analysis was undertaken on the basis of methodological quality and none of the studies were eliminated. 

### 2.5. Meta-Analysis

Studies which involved the comparison of a dietary measure (i.e., FR) to energy expenditure as measured by doubly labelled water (DLW) were sufficiently similar methodologically to enable meta-analysis to be performed. To quantitatively compare differences between reported and measured energy intake in the papers using DLW, the between-group standardised mean difference, or effect size (ES), and a 95% confidence interval (CI) was calculated. The extracted data (i.e., mean energy intake, mean energy expenditure, SD, and sample size) was transferred into Comprehensive Meta-Analysis (CMA) version 2 software (Biostat, 2005, Englewood, IL, USA) in order to calculate the ES, standard error, variance, and 95% CIs. A forest plot was generated to display the ES and 95% CIs from each study and the pooled estimate determined, whereby a small ES was > 0.2, a medium ES > 0.5, and a large ES > 0.8 [39]. A positive ES indicated an effect favouring energy measured by DLW while a negative ES indicated an effect favouring reported EI.

## 3. Results

### 3.1. Identification and Selection of Studies

**Figure 1:**
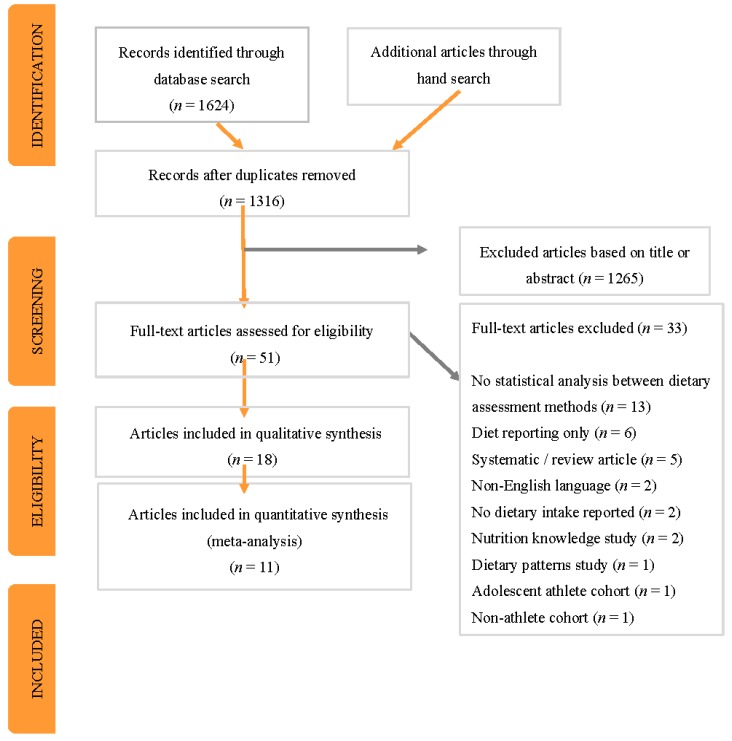
Preferred Reporting Items for Systematic Reviews and Meta-Analyses (PRISMA) flow diagram of identification, screening and selection process for included articles.

The initial literature search identified 1624 potentially relevant papers. After the removal of duplicates and the elimination of papers based on title and abstract, 42 full text publications were reviewed in duplicate against the inclusion criteria. A manual search of reference lists identified an additional nine papers. Thirty-three papers were excluded because they did not meet the inclusion criteria, leaving 18 full-text articles for assessment (Figure 1).

### 3.2. Demographic and Anthropometric Characteristics

Of the 18 studies included, 11 compared reported energy intake (EI) against energy expenditure (TEE), as measured by DLW (TEE_DLW_) [21,22,40,41,42,43,44,45,46,47,48]. The remaining seven papers [29,49,50,51,52,53,54] compared a new dietary tool (*n* = 3) or Food Frequency Questionnaire (FFQ) or modified FFQ (*n* = 4) to a reference method(s) (i.e., food record, 24-h dietary recall, direct observation, biomarker) as part of a validation study. Of the 18 included studies, three compared one or more dietary assessment method(s) to a biomarker (i.e., urine *n* = 2, blood *n* = 1) [45,50,54].

The total number of study participants was 683 (range 7–156 participants); with smaller sample sizes noted in papers comparing energy intake to DLW (range 7–19 participants). A range of sports were represented, most of which were ‘mixed sports’ (6 studies; *n* = 461 participants) with the remainder reporting on athletes from ‘aerobic’ or ‘endurance sports’ (e.g., long distance running, swimming, rowing, triathlon, cross country skiing) (nine studies; *n* = 184), ‘team sports’ (e.g., soccer, basketball) (two studies; *n* = 26), and ‘skill’ or ‘aesthetic sports’ (one paper; *n* = 12 ballet dancers). The mean age was 21.8 ± 2.6 years (range 16–30.4 years). Two studies did not report participant age, however it was assumed the participants were older than 16 years of age due to recruitment from tertiary institutions [41,53]. More than half of the studies were in male athletes (55% participants). Athletic calibre included competitive tertiary, national, international, and professional level athletes. The studies were conducted in Europe (*n* = 6), North America (*n* = 6), and across the Asia Pacific and African regions (*n* = 6) (Table 1).

Physique characteristics of the athletes are summarized in Table 1. The weighted mean body mass and stature were 65.3 ± 7.2 kg and 173.3 ± 6.1 cm, respectively. One paper did not report the mean stature of participants [50]. Seven studies reported percentage body fat with mean levels ranging from 7.1 ± 2.5 to 22.8 ± 5.1% (Table 1).

### 3.3. Studies Comparing Reported Energy Intake to Energy Expenditure as Measured by DLW

**Figure 2:**
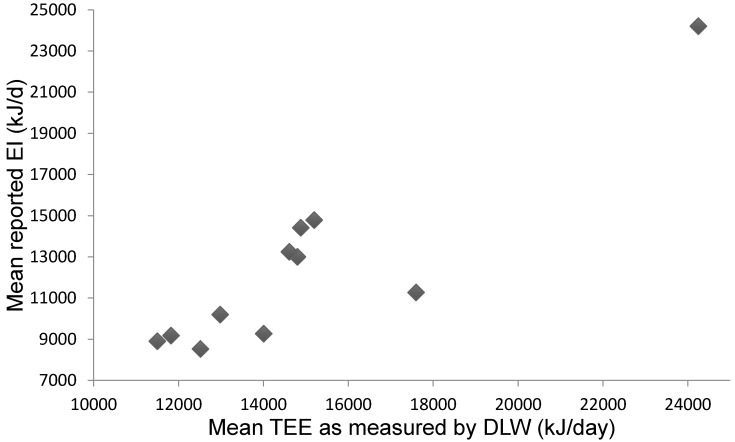
Mean reported energy intake (EI) when compared to mean energy expenditure (TEE) measured by doubly labelled water (DLW) (kJ/day).

Eleven studies assessed the difference between reported mean EI and mean TEE, as measured by DLW (Figure 2). The most common method for measuring EI was by weighed or estimated FR over 4–10 days (mean 6.5 days) duration (Table 2). Where reported (*n* = 5), the mean macronutrient intake (as percent of EI) was 56.5, 14.9, and 26.8% for carbohydrate, protein, and fat, respectively. One paper reported mean protein intake only (12.6% of energy intake) [45].

Energy expenditure was measured for a mean of 8.4 days (range 6–14 days) by the DLW method (Table 2). Details on the training phase or competitive season of the participants were reported by six studies [21,22,40,42,43,47]. Six studies also recorded the physical activity levels during the assessment period, either by self-reporting the number of training hours via an activity record (*n* = 5) [22,41,45,46,48], or as measured by ActiGraph™ activity monitor (*n* = 1) [42]. 

Eight of the eleven studies measured dietary intake for the entire DLW measurement period [21,22,40,41,42,45,46,47]; while, three studies measured intake for only 4–5 days [43,44,48]. Most of the studies (*n* = 7) reported a stable body mass during the testing period, however four studies adjusted energy for changes noted in body mass during the testing period [43,44,45], or for poor dietary records as a result of participant non-compliance [22] (Table 2).

Resting metabolic rate (RMR) was measured via indirect calorimetry in four studies [40,43,46,47]; four reported RMR based on prediction calculations (i.e., Schofield, Cunningham or FAO/WHO/UNU equations) [21,42,44,48]; while, three studies did not report RMR [22,41,45] (Table 2).

#### Mean Difference between EI and TEE: Reporting Bias

When compared to DLW, all studies reported a lower mean EI of 19% (range 0.4–36%), with a weighted mean difference of −2793 ± 1134 kJ/day between measures (Table 3). The difference was significant in seven studies [21,40,41,42,44,45,47], while smaller, non-significant differences were reported in the remaining studies [22,43,46,48]. For example, Hill and Davies [44] found a very strong inverse correlation of difference between TEE and self-reported EI, and the means of the two measurements (*r* = −0.93, *p* < 0.01) in elite female lightweight rowers. When mean EI was adjusted for change in body mass (−1.2 ± 1.2 kg) over the measurement period, the correlation of difference between the mean adjusted TEE and self-reported EI was also significant (*r* = −0.93, *p* < 0.01). The limits of agreement (LOA) (95% CI ± 2 SD) for the mean adjusted TEE and self-reported EI were −17,619 to 8134 kJ. The authors also noted that reporting bias increased in those with higher TEE (i.e., TEE > 3000 kcal/day; or 12,552 kJ/day). In contrast, Schulz and colleagues [46] found no relationship between self-reported EI and TEE in female distance runners, and reported a mean difference of −925 ± 2301 kJ/day. Similarly, Sjodin and colleagues [48] noted a close match between reported EI and TEE (i.e., mean difference of —100 ± 1900 kJ/day) in eight cross-country skiers (Table 2). 

Koehler and colleagues [45] aimed to assess the validity of a food and activity record as compared to reference methods, such as DLW, 24-h urea nitrogen excretion, and indirect calorimetry (i.e., to determine energy expenditure during an incremental running or cycling test). They found EI and TEE as measured by DLW were only weakly correlated, however following the exclusion of two implausible results (where EI < 1.39 × REE) [55] a significant positive correlation was observed (*r* = 0.69, *p* < 0.05). A proportional bias towards over-estimating low and under-estimating high energy intakes (*p* < 0.01) was also noted with very wide LOA (95% ± 2 SD) between EI and TEE of −5736 and 4912 kJ/day (Table 2).

### 3.4. Meta-Analysis

**Figure 3:**
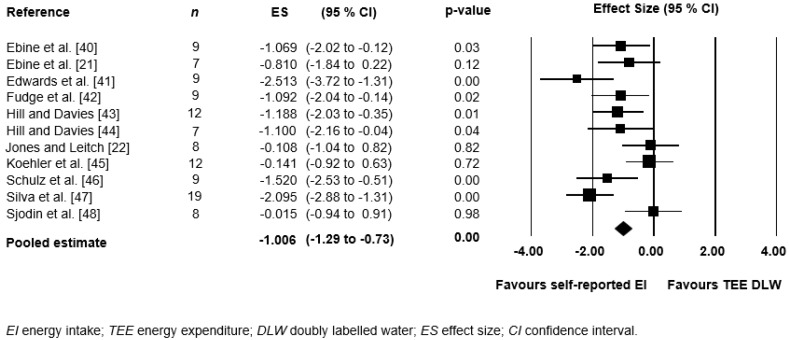
Meta-analysis of the pooled effect of self-reported EI when compared to TEE measured by DLW.

Studies that involved the comparison of a dietary measure (i.e., self-reported or weighted FR) to TEE assessed by the DLW technique were homogenous enough to conduct a meta-analysis (*n* = 11). Relevant data (i.e., mean EI, SD, and sample size) were used to calculate the between-group standardised mean difference, or effect size (ES), and 95% confidence intervals (CIs). The meta-analysis revealed a large pooled ES of −1.006 (95% CI −1.3 to −0.7; *p* = 0.00) where reported EI was significantly below TEE measured by DLW (mean −2477 kJ/day) (Figure 3).

### 3.5. Studies Comparing Reported Dietary Intake by Two or More Methods of Dietary Assessment

Of the seven studies comparing reported intake by two or more methods of dietary assessment, four compared energy and/or macronutrient intake [49,51,52,53], while the remaining three studies compared the intake of a specific nutrient (i.e., antioxidants, calcium, or protein) [29,50,54]. Only two studies validated the test method (i.e., FFQ, modified FFQ) for micronutrients and food groups [51,53] (Table 4).

Four studies investigated the validity of a new self-administered, semi-quantitative, or quantitative food questionnaire (i.e., FFQ or modified FFQ) in an athlete cohort. The dietary reference methods that were used to evaluate these new instruments included a FR (*n* = 3) [29,50,51] or multiple pass 24-h dietary recall (DR) (*n* = 1) [53]. Braakhuis and colleagues [50] compared a blood biomarker of antioxidant capacity (i.e., ferric-reducing ability of plasma [FRAP]) with antioxidant intakes that were recorded from a 7-d weighed FR and FFQ. The remaining three studies aimed to validate a new dietary assessment instrument (e.g., web-based 24-h DR, virtual interface tool). The dietary reference methods that were used to evaluate these new instruments included a FR (*n* = 1) [52]; multiple pass 24-h DR (*n* = 2) [49,54]; and/or, direct observation (*n* = 1) [49]. Wardenaar and colleagues [54] also compared self-reported protein intake, as measured by 24-h DR to urinary nitrogen excretion (Table 4).

#### 3.5.1. Reported Mean Energy Intake

Six of the seven studies reported mean EI and/or compared intake between dietary assessment methods (Table 4). Sunami and colleagues [53] compared mean EI of college athletes from a variety of sports from a FFQ (138 items), with 3-d non-consecutive 24-h DR and found the FFQ under-estimated EI by 9% (males) and 10% (females). In comparison, Fogelholm and Lahti-Koski [51] found close agreement on a group level of mean EI of mixed sport athletes from a food use questionnaire (FUQ) as compared to a 7-day weighed FR (95% CI −1.7, 0.1 FUQ1, 95% CI −0.1, 1.7 FUQ2); however, individual agreement was weak. Scoffier and colleagues [52] found no difference between EI as measured by a virtual interface dietary assessment tool when compared to a 1-d FR in two groups (i.e., weight sports, and other mixed sports) of adolescent athletes. Baker and colleagues [49] compared reported energy and macronutrient intakes measured by a web-based, 24-h DR tool (DATA), to a 24-h DR (interview) and observation (by registered dietitian), and found no significant difference between the measures. They found good relative validity for group level comparisons, but large variations of individual dietary intake estimates, especially in athletes with higher energy and macronutrient intakes (Table 4).

#### 3.5.2. Reported Mean Macronutrient Intake

Where reported (4 of 7 studies), the mean macronutrient intake (as percent of EI) was 53.1, 15.3, and 28.5% for carbohydrate, protein, and fat, respectively (Table 4). An additional study reported a mean protein intake of only (11.9% of EI) [54]. Wardenaar and colleagues [54] reported a mean difference between 24-h DR and 24-h nitrogen excretion (25.5 ± 21.3% difference, or 31.7 ± 30 g/day; *p* < 0.001). Under-reporting of protein was related to the amount of protein intake (*r* = −0.20; 95% CI −0.46, 0.09) such that under-reporting was greater at a higher protein when compared to a lower protein intake. In contrast, Koehler and colleagues [45] found that protein intake in triathletes was strongly correlated to 24-h urinary nitrogen excretion (*r* = 0.81; SEE = 0.34 g/kg/day), however considerable individual variation was observed between the two methods with very wide limits of agreement (−0.65 to 0.67 g/kg/day).

#### 3.5.3. Other Nutrients, Food Groups and Dietary Patterns Reported

Braakhuis and colleagues [50] found a modest correlation between energy-adjusted estimates of total antioxidant intakes in competitive rowers, as measured by a quantitative FFQ (70 items) when compared to 7-d weighed FR (*r* = 0.38, 90% CI ± 0.14), and only a small correlation between the FFQ and plasma biomarker (*r* = 0.28, 90% CI ± 0.15). However, the authors noted a trend to over or under-estimate antioxidant intakes at low and high intake levels (42% over and 73% under-estimation, respectively). Ward and colleagues [29] compared mean calcium intake, as measured by a self-administered calcium checklist to a 6-day FR (822 ± 331 mg/day and 823 ± 387 mg/day, respectively) and observed no significant difference between the two methods (Table 4).

In addition to comparing energy and macronutrient intake between the test method (i.e., FUQ1 and FUQ2) and a 7-day weighed FR, Folgelholm and Lahti-Koski [51] reported on selected micronutrients (i.e., thiamin, vitamin C, calcium, magnesium, iron, and zinc). They found the FUQ1 over-estimated the reported intake of vitamin C, calcium, magnesium, iron, and zinc; while, the FUQ2 did not differ in mean values for the selected micronutrients when compared to a 7-d FR. Most of the correlations by food group were reported above *r* = 0.24, with the exception of ‘vegetable oils’, ‘cream’, ‘milk’, ‘pork’, ‘beef’, and ‘poultry’ [51].

One study validated the test dietary assessment method (i.e., FFQ) as compared to a 3-d non-consecutive 24-h DR for 35 nutrients and 19 food groups [53]. While the difference in measures for the majority of nutrients was within ± 20%, the largest difference was observed for retinol (i.e., 77% for males and 32% for females). The authors found lower differences between the measures for ‘cereals’ (1% in males, 10% in females), ‘vegetables’ (9% in males, 4% in females), and ‘fungi’ (5% in males, 9% in females) food items; while greater differences were noted for ‘sugar’ (−94% in males, −96% in females), ‘beverages’ (−45% males, −54% females), and ‘seasonings and spices’ (−65% males, −72% females) food items. Overall, under and over-estimation of food group intakes was more prevalent than that of nutrient intakes [53] (Table 4).

### 3.6. Evaluation of Methodological Quality

The methodological quality of all the included studies (*n* = 18) were evaluated with quality ratings that were determined as fair to moderate for the majority of studies (Table 5). All of the papers stated the aim or hypothesis, described the main outcomes to be measured, described the main findings, reported clinical and statistical significance, and accounted for participant drop outs. All but two studies provided estimates of the random variability [43,46]; reported on data dredging [44,48]; and all but two reported sources of funding [44,45]. However, the poorest ratings were for the following items: “Were actual probabilities reported?”(Item 7); “Were the subjects asked to participate representative of the entire population?” (Item 10); “Were the participating subjects representative of the population they were recruited from?” (Item 11); and “Were the subjects recruited over the same time period?” (Item 18) Only two studies [49,54] reported whether an attempt was made to blind those measuring the main outcomes of the intervention (i.e., Item 12) (Table 5).

## 4. Discussion

This is the first systematic review to have evaluated studies comparing two or more dietary assessment methods, including measuring intake against biomarkers or reference measures of energy expenditure, in athletes. Only 18 papers were published over the past 37 years, highlighting the limited literature in this area. Most papers (*n* = 11) focused on self-reported EI as compared to TEE, as measured by the DLW technique [21,22,40,41,42,43,44,45,46,47,48]. The remaining studies (*n* = 7) compared a dietary assessment method to a reference method(s) (e.g., food record, 24-h DR, direct observation, biomarker) as part of a validation study [29,49,50,51,52,53,54]. Dietary assessment methods that are recognized as appropriate for the general population are usually applied in a similar manner to athlete groups, despite the knowledge that athlete-specific factors can complicate the assessment and impact accuracy in unique ways. As dietary assessment is used extensively in both sports nutrition clinical and research settings, it is a concern that the validity of the methodologies used have not undergone more rigorous evaluation. There is a clear need for high quality research to be undertaken to identify dietary assessment methodologies which are valid, as well as feasible for use, in an athlete population.

### 4.1. Studies Comparing EI to TEE as Measured by DLW 

Overall, the studies (*n* = 11) comparing self-reported EI to TEE_DLW_ found that mean EI was under-estimated by 19% (range 0.4–36%), which is comparable to differences observed in studies in other populations [18,19,20,25,26,56,57]. For example, in a study involving obese participants, Schoeller and colleagues [58] reported up to a 30% difference between measures. Three DLW studies [22,45,48] reported close agreement (<5% difference between measures); which can be partially explained as participants were provided with a test diet for the duration of the DLW collection and a participant was excluded due to an inexplicable marked difference in agreement between measures [22]. Koehler and colleagues [45] adjusted mean EI following the removal of participants (*n* = 2) for loss of body mass (>3%) or due to implausible food records, as determined by a cut-off value (i.e., EI < 1.39 × RMR) [55]. While Sjodin and colleagues [48] noted a close agreement between mean EI and TEE_DLW_ over seven days (*r* = 0.96; *p* = 0.0001), no relationship was observed when authors compared EI from separate 24-h periods, indicating that athletes were not in energy balance during shorter periods of time.

The remainder of the DLW studies (*n* = 8) reported a greater mismatch between self-reported EI and TEE_DLW_ (12–34% difference) attributed primarily to misreporting [21,40,41,42,43,44,46,47]. A variety of explanations have been proposed for lower reported EI when compared to TEE_DLW_ [21,25,40,46]. Hill and Davies [18] suggest factors such as body size, perception of body image, restrained eating, gender, socioeconomic status, motivation, social expectations, and the nature of the testing environment itself, play a role in misreporting. Nutrition-related beliefs and dietary practices of athletes present additional challenges with reporting intake accurately in this population sub-group. In addition, factors such as high energy requirements, frequency of snacking, eating away from the home environment, applicability of ‘standard’ portion sizes, and the wide use of commercial sports foods, drinks and supplements can make it more difficult to quantify food intake [8,19].

#### 4.1.1. Methodological Issues

The DLW technique has long been considered a ‘gold standard’ for measuring TEE and to validate self-reported EI [20]. While the DLW technique is considered accurate to 1%, with a coefficient of variation of 2–12%, depending on the loading dose and length of the sampling period [20,58]; potential sources of error should be considered when measuring in free-living conditions, including the influence of day-to-day variation of EI and TEE [58]. The optimal duration for measuring DLW is between 2–3 biological half-lives of the isotopes [59]; although a shorter period (i.e., 8–12 days) has been suggested for an athlete cohort due to higher rates of water turnover due to regular physical activity [59]. A potential limitation is the DLW measurement period of most of the included studies (*n* = 8) may have been too short (i.e., 6–7 days duration) [21,40,41,42,45,46,47,48].

#### 4.1.2. Assessment of Dietary Intake

Typically, dietary intake is measured in DLW studies to assist in the calculation of the respiratory quotient (RQ); a required variable in calorimetry equations [60]. This calculation was reported in the methodology of only four of the included studies [22,40,46,47]. In non-athletes, a 3–4 day FR is considered as valid for the assessment of RQ and EI for groups [61]; however, dietary intake of athletes may be more variable due to the day-to-day variation in the energy cost of training. It has been suggested that recording intake for 3–7 days is a reasonable compromise between scientific rigor and practicality when estimating dietary intake of athletes to capture habitual intake and high variability in day-to-day energy expenditure [19,28,62]. Hence, for athletes, ideally intake would be recorded over the entire DLW measurement period to capture this variation. As such, a further limitation is that three studies did not record intake for the same duration as the DLW measurement [43,44,48]. 

In all of the included DLW studies, dietary intake was determined by weighed or estimated FR recorded between 4–10 days. The documentation of intake via FR is considered practical and it is widely used in both clinical and research settings, however the accuracy of data is reliant on subjective recording of intake, level of participant motivation, and possible fatigue from recording for longer durations [18,19,25]. Recording intake itself can modify usual eating behavior [46,63]. Investigators are responsible for improving compliance by providing clear, specific instructions to ensure records provide sufficient detail. Nine studies provided detail about the instructions provided to participants and the review process of dietary records upon collection [21,40,41,42,43,44,46,47,48]. Only one study supervised the weighed FR over a limited observation period in order to improve recording accuracy [48]; while another provided a test diet to all of the participants [22]. 

Given the potential for misreporting during the recording period, the plausibility of self-reported EI can be compared to a pre-determined EI: BMR value [55]. Two DLW studies applied a cut-off value to exclude implausible dietary records [44,45], and one of the validation studies [50]. However, when applying a cut-off value to assess self-reported EI of athletes, it is important to have a valid assessment of the energy demands of the athletes being assessed, as the range in TEE can be wide and is reliant on the type of sport, training phase, and intensity [19,56,64].

#### 4.1.3. Variability of Intake and Expenditure in Athletes

Although the DLW studies investigated energy balance or the misreporting of intake, this was not the primary aim of all the included papers. A number of studies (*n* = 4) aimed to determine TEE of a group of athletes (i.e., swimmers, soccer players, light weight rowers, distance runners) and compare to recommendations [21,40,44,46]; with the secondary outcome to evaluate the agreement between reported EI and TEE_DLW_. The primary aim of the remaining papers (*n* = 7) was to compare the difference between EI and TEE_DLW_ [22,41,45,47]; assess energy balance in a group of athletes (i.e., endurance runners, cross-country skiers) [42,48]; and, assess the validity of a 4d FR in measuring EI of female classical ballet dancers [43].

While DLW is highly regarded as an accurate method for the validation of reported EI [64], a major limitation is that the technique is unable to calculate energy turnover on a daily basis. In athletes, TEE (and EI) can fluctuate substantially from day-to-day [65,66]. For example, Bradley and colleagues [65] found the EI of rugby union players lowest when TEE was highest, early in the training week, with EI increasing in preparation for competition on the weekend. Similarly, Brown and colleagues [66] noted differences in EI, macronutrient intake, and energy balance of dancers between weekdays and weekend days. These recent publications highlight the day-to-day variability in energy demands of athletes over a relatively short period of time, which is unable to be assessed by DLW.

#### 4.1.4. Influence of Body Mass, Body Image, and Energy Demands

In most studies, there was no significant change in body mass during the assessment period [21,22,40,41,45,46,47,48], indicating that the difference between measures was likely due to misreporting. Conversely, Fudge and colleagues [42] attributed the difference that was noted between EI and TEE_DLW_ (−13%) in elite Kenyan runners to under-eating, resulting in a negative energy balance during a period of intense training. Four studies adjusted mean TEE_DLW_ based on changes in body mass during the measurement period [22,43,44,46]. Previous research has indicated that the degree of misreporting increases with increasing body mass, specifically adiposity [67,68] and BMI (body mass index) [69]. Others suggest that misreporting is independent of adiposity, but is linked to restrained eating, and body image [18,70,71,72]. In the current review, Edwards and colleagues [41] observed a relationship between EI and body mass (*r* = −0.74) (i.e., heavier women reported a lower relative intake). However, Hill and Davies [43] found no relationship between the extent of misreporting and percent body fat of female ballet dancers (*r* = 0.11). Similarly, Silva and colleagues [47] found no relationship between EI and body mass or composition in male and female basketball players. 

It appears that the trend for misreporting increases with an increasing TEE, particularly for individuals with high energy needs [19,57]. Possible reasons include increased burden from reporting large volumes of food and frequent eating occasions resulting in food or drink items being omitted consciously or unconsciously. In the current review, a negative correlation was observed between self-reported EI and TEE_DLW_ in some studies (*r* = −0.854, *p* < 0.01) (i.e., the higher the expenditure, the lower the reported intake) [40,41]. While there was almost no difference between self-reported EI and TEE_DLW_ in a group of triathletes, the data indicated a strong proportional bias towards under-estimating high energy intakes (*p* < 0.01) [45]. Similarly, Hill and Davies [44] reported a bias to misreporting with an increasing EE (i.e., TEE > 3000 kcal/day; or 12,552 kJ/day) in female lightweight rowers. This trend is supported by findings in non-athletes where the magnitude of misreporting intake increases with increasing TEE [57,73,74].

### 4.2. Studies Comparing Dietary Intake by Two or More Methods of Dietary Assessment 

The current review identified a limited number of studies (*n* = 7) that evaluated the validity of a novel dietary assessment tool or instrument when compared to one or more reference method(s) [29,49,50,51,52,53] and/or a biomarker in an athlete population [50,54]. However, due to limited consistency in the pairing of study methods, it was difficult to make firm conclusions on the relative strength of the different methodologies that are used to evaluate dietary intake in athletes. Despite most studies reporting an acceptable validity for group level comparisons [49,51,54], individual agreement was not as strong, especially for athletes with high energy intakes [49,50,54]. A variety of statistical tests were used to assess the study validity (e.g., paired t-tests, correlation coefficients, Bland-Altman plots), however most studies used correlation coefficients [50,53] or intra-class correlation (ICC) to assess reproducibility [29,49]. Correlation coefficients can be misleading, as they measure the relationship between two methods, rather than the agreement between them [75]. Only one study validated the test method for food groups [53], while the other studies evaluated energy and/or nutrient intakes [29,49,50,51,52,54] 

#### 4.2.1. Dietary Reference Methods

Three of the included studies determined dietary intake by weighed or estimated FR recorded between 6–7 days [29,50,51]; while another study recorded intake for a single day only [52]. Research suggests that a longer recording duration is necessary to assess habitual intake and account for day-to-day variation of intake and TEE in athletes, with a 7-d FR two to three times less variable than a 1-d FR [28]. Other reference methods that rely on self-reporting (e.g., 24-h DR, FFQ) are also prone to measurement error associated with recall bias and awareness of portion size [19,53,76]. Three studies used the multiple pass 24-h DR [49,53,54] and/or direct observation [49] to evaluate a new tool or instrument. While 24-h DR has the advantage of low participant burden, data may not be representative of the usual diet unless the recalls are repeated a number of times [4,19]. In addition, Wardenaar and colleagues [54] suggest that dietary data should be collected within a four week period to provide the best insight into the accuracy of the multiple 24-h DR in athletes, due to the micro-cycle and periodization of training (i.e., training programs with a variable workload and volume throughout the year). It has been previously suggested that the 24-h DR is prone to underestimate EI, and that caution should be taken assessing data involving high intakes [4,19,77]. In general, all dietary assessment methods are influenced by errors of precision and validity [4,67]. As such, it is possible that the validation studies included in the current review failed to detect true reporting bias, if both the new and established dietary assessment instruments have correlated error [20], particularly when they are applied to an athlete population. Researchers are encouraged to consider the relative strengths and weaknesses of the dietary reference method(s) selected, in addition to consider participant burden, cost, and validity in the study population [53].

#### 4.2.2. Evaluation Using Biomarkers

Two of the included validation studies used a biomarker to assess the accuracy of reported intake [50,54]. Wardenaar and colleagues [54] found a multiple 24-h DR acceptable for ranking protein intake compared to the reference urinary nitrogen biomarker (*r* = 0.65). However, they noted that the ‘standard’ portion sizes used in the 24-h DR may not be representative for athletes with high intakes of protein, and therefore a potential source of error [54]. Braakhuis and colleagues [50] found only a weak association between the plasma antioxidant biomarker (i.e., FRAP) and FFQ (*r* = 0.28). However, relying on blood biomarkers to validate a nutrition questionnaire can be problematic as there is no single marker for antioxidant intake [50]. Unfortunately, the use of independent biomarkers to assess the accuracy of dietary intake is limited to energy intake or a specific nutrient only (i.e., sodium, nitrogen). Koehler and colleagues [45] indicate that their research first to validate dietary protein intake against 24-h urinary nitrogen excretion in an athlete population. They found good agreement between 24-h nitrogen excretion and dietary protein intake (*r* = 0.81), which is consistent with the results documented in non-athlete populations [56,64,76]. More recently, energy availability (EA) (i.e., EA = EI − ExEE) has been linked to biochemical indices, such as cortisol, insulin, growth hormone, IGF-1, leptin, and thyroid hormones [45,78]. These present potential use as biomarkers in future dietary assessment studies.

#### 4.2.3. Nutrients, Food Groups and Dietary Patterns

Micronutrient intake and food group intake is less frequently reported in dietary validation studies than energy and macronutrient intakes [38]. One paper evaluated calcium intake in female college athletes from a range of sports (e.g., basketball, cross-country) using a 6-d weighed FR to validate a self-administered calcium checklist [29]. Mean calcium estimates did not differ between measures, however longer periods of recording may be required to estimate the intake of key micronutrients, such as iron and calcium (i.e., up to 11 recording days) [19,61,62,77]. In another study, Braakhuis and colleagues [50] found that an FFQ was valid for estimating antioxidant intake in elite rowers. 

Two studies reported on a range of selected micronutrients [51,53] as part of validating test methods (i.e., FUQ, FFQ, respectively). These same studies are the only studies in athletes to validate dietary intake at the food group level. Sunami and colleagues [53] found a FFQ was useful for assessing habitual dietary intake of college athletes for vitamin C, calcium, vegetables, fruits, and milk and dairy products. While Fogelholm and Lahti-Kosti [51] found close agreement of EI between the FR and FUQs, with most food group correlations above *r* = 0.24, except for ‘vegetable oils’, ‘other fats’, ‘standard milk’, ‘pork’, ‘beef’, and ‘poultry’. 

More recently, nutrition epidemiology has progressed from examining nutrients per se to exploring the relationship of dietary patterns or diet quality on health outcomes [79,80]. However, to date, there has been limited evaluation of diet quality or dietary patterns in athletes [8,81,82,83]. Dietary patterns, including the specific timing of intake over the day, have been shown to enhance health, training, and performance outcomes [1,83], and should be addressed in future studies. Acknowledging that there are challenges with assessing intake accurately, it may prove beneficial in understanding dietary patterns or diet quality of athletes to help identify athletes who may benefit from dietary input, and provide a platform to educate individuals about dietary choices for optimal health and sports performance.

### 4.3. Qualitative Assessment of Methodological Quality

Overall, the studies that were included in this review (*n* = 18) were of fair to moderate quality. Poor ratings were generally noted for items that evaluated external validity and internal validity. For example, selection criteria (i.e., inclusion and exclusion criteria) and representativeness of the participants was usually not adequately described. Ten studies included an adequate description of participant characteristics (i.e., item 3) [21,22,40,41,42,46,47,49,50,54]; while, only six studies [21,29,42,50,51,54] clearly identified the source and how participants were recruited (i.e., item 10) and/or stated the proportion who were asked agreed to participate in the study (i.e., item 11). Potential confounding factors, such as supplement use, maintenance of body mass, or maintenance of usual physical activity levels, were often not discussed. The description of and adjustment for confounding factors (i.e., items 4 and 19, respectively) were either unable to be determined or deemed not relevant for most of the DLW papers [21,22,40,41,44,45,46,47,48] due to the nature of methodology involving DLW studies. Finally, attempts that were made to blind investigators to the main outcomes of the intervention were poorly rated for all but two studies [49,54]. Despite known challenges with reporting dietary intake accurately using FR; monitoring of and compliance with self-reported intake data was not always considered in the included studies. Twelve studies indicated that the expertise of qualified dietitians were involved in the collection and/or or analysis of dietary data [22,41,42,43,44,46,47,48,49,50,51,54]. 

## 5. Limitations, Strengths and Future Directions 

One of the key strengths of the current review includes the extensive, systematic search, and the evaluation of the literature, which has compared two or more dietary assessment methods, including measuring intake against dietary biomarkers or reference measures of energy expenditure in athletes. The results indicate that there are limited robust studies evaluating the validity of dietary assessment methods in athletes. The calibre of participants could have influenced study findings and relevance for an elite athlete population. For example, a number of studies involved participants from university sporting clubs or teams [22,29,41,43,49,53], while two studies did not provide sufficient detail about the recruitment source or participant athletic calibre [45,52]. The relatively small sample size in all but two of the eighteen papers could influence the power that is required to detect meaningful differences. For validation studies, Serra-Majem and colleagues [38] suggest including a sample size that is greater than 100 participants, or more than 50 participants when using a biomarker. However, it is not uncommon for sports science research to include a relatively small population sample, which could make it difficult to detect significant change [84]. There may also be challenges in recruiting a sample that is powerful enough and representative of high level athletes due to the reticence of athletes to commit to research studies perceived as time consuming and detracting from training or competition commitments. A further consideration is that the isotope deuterium that is used in DLW studies is expensive, which could influence sample size due to cost and research feasibility.

It has been suggested that combining two or more methods of dietary assessment may enhance the accuracy of assessing dietary intake [85]. For example, Rumbold and colleagues [86] found that a combination of FR and 24-h DRs were effective when quantifying EI in adolescent netball players (i.e., 4.2% difference between measures indicating a slight bias towards over-reporting). In a similar study protocol, Briggs and colleagues [87] compared the accuracy of a combined dietary data collection method (i.e., weighed FR and 24-h DR) to the observed EI of adolescent male soccer players. The results showed systematic under-reporting of intake as compared to observed intake (*p* < 0.01), but the bias was small (i.e., degree of random error between dietary methods 3.1%). These publications suggest a combined dietary data collection method may provide an effective technique when quantifying energy intake in athletes.

Finally, emerging image-assisted technical innovations such as wearable cameras, handheld devices, and mobile telephone technology have been shown to improve participant compliance by reducing the burden of recording and enhance the accuracy of data recorded [30,31,32,33]. Results from a systematic review by Gemming and colleagues [32] indicate that images enhance self-reporting by revealing unreported foods and identifying misreporting errors that are not captured by traditional assessment methods alone. The use of an image-based FR deployed via a mobile application has recently been shown to be a positive tool for dietary monitoring and potential influence on dietary habits and behaviors [88].

## 6. Conclusions

Adequate dietary intake is crucial for the maintenance of health and optimizing performance outcomes of athletes. However, there are unique challenges in assessing intake, including the day-to-day variation of expenditure and wide use of commercial sports foods and supplements, which can challenge the accuracy of dietary assessment methods that are used in athletes. Results from this review suggest that self-reported food records may not be a suitable assessment method for quantifying energy expenditure, particularly for weight conscious athletes or athletes with high energy demands. Existing literature demonstrates substantial variability between dietary assessment methods, with under and misreporting of intake frequently observed. As such, there is a clear need for careful validation of dietary assessment methods with emerging technical innovations being likely to show promise as they may assist with portion quantification, reduce the burden of collection, and problems with missing foods, among athlete populations.

## Figures and Tables

**Table 1:** Participant characteristics of athletes from included studies.

Reference, Country	Group (*n*)	Sport, Calibre	Age (Years)	Body Mass (kg)	Stature (cm)	BMI (kg/m^2^)	Body Fat (%)	Comments
Baker et al. (2014), USA [49]	56 (41 M, 15 F)	Mixed sports ^1^, competitive, tertiary	16 ± 2 (14–20)	69.4 ± 14.3	174.3 ± 9.4			*n* = 12 excluded (non-adherence)
Braakhuis et al. (2011), USA [50]	113 (56 M, 57 F)	Rowers, national competitive	22 ± 3 (17–36)	78.0 ± 11.0	NR			*n* = 2 excluded (EI < 1.39 * RMR)
Ebine et al. (2000), Japan [40]	9 F	Synchronised swimmers, national	19.8 ± 2.8 (16–23)	52.5 ± 2.7	159.0 ± 3.0	20.7 ± 0.7		DLW
Ebine et al. (2002), Japan [21]	7 M	Soccer, professional	22.1 ± 1.9	69.8 ± 4.7	175.0 ± 5.0		13.4 ± 3.6	DLW
Edwards et al. (1993), USA [41]	9 F	Cross-country runners, highly trained, tertiary	NR #	55.3 ± 6.2	169.1 ± 5.5	19.3 ± 1.7	13.0 ± 3.2	DLW
Fogelholm & Lahti-Koski (1991), Finland [51]	84 M	Mixed sports ^2^, recreational	24 ± 4	72.0 ± 7.0	180.0 ± 6.0			*n* = 12 excluded (incomplete data)
Fudge et al. (2006), Kenya [42]	9 M	Middle & distance runners, elite	21 ± 2	56.0 ± 3.4	174.0 ± 2.9	18.3 ± 1.3	7.1 ± 2.5 (BIA)	DLW
Hill and Davies (1999), Australia [43]	12 F	Ballet dancers, tertiary	18.7 ± 1.2	57.3 ± 3.8	169.1 ± 4.6			DLW
Hill and Davies (2002), Australia [44]	7 F	Lightweight rowers, elite	20.0 ± 1.1	60.9 ± 2.3	168.8 ± 4.7		22.8 ± 5.1	DLW *n* = 1 excluded (incomplete data)
Jones and Leitch (1993), Canada [22]	8 (5 M, 3 F)	Swimmers, tertiary	20.1 ± 1.7	74.1 ± 9.3	186.0 ± 11.0		16.0 ± 6.4	DLW *n* = 1 excluded (disagreement between measures)
Koehler et al. (2010), Germany [45]	12 M	Triathletes, well-trained ^a^	30.4 ± 6.2	80.6 ± 6.5	186.0 ± 8.0	23.2 ± 1.4	11.2 ± 2.1(BIA)	DLW *n* = 2 excluded (change BW > 3%; EI < 1.39 * RMR)
Schulz et al. (1992), USA [46]	9 F	Distance runners, elite, national	26.0 ± 3.3	52.4 ± 4.1	163.0 ± 7.0		12 ± 3 (UWW) 17 ± 3 (BIA)	DLW
Scoffier et al. (2013), France ^ [52]	22 (13 F, 9 M) 20 (12 F, 8 M)	Mixed sports ^a^ Adolescent, weight sports Adolescent, other sports	25.3 ± 4.7 26.4 ± 5.7	59.7 ± 15.7 * 62.1 ± 10.7 *	169.8 ± 2.1 * 168.8 ± 2.7 *			
Silva et al. (2013), Portugal [47]	19 (12 M, 7 F)	Basketball, junior national, elite	17.0 ± 0.7 (M) 16.9 ± 0.7 (F) (16–18)	74.7 ± 10.8	185.4 ± 11.0	21.7 ± 1.9		DLW
Sjodin et al. (1994), Sweden [48]	8 (4 M, 4 F)	Cross-country skiers, elite, national	26 ± 2 (M) 25 ± 2 (F)	75.1 ± 4.9 (M) 54.4 ± 5.1 (F)	180.0 ± 6.0 (M) 166.0 ± 2.0 (F)			DLW
Sunami et al. (2016), Japan [53]	156 (92 M, 64 F)	Mixed sports ^3^, tertiary	NR#	68.7 ± 8.4 (M) 56.1 ± 5.9 (F)	174.7 ± 6.5 (M) 163.4 ± 5.8 (F)	22.5 ± 2.1 (M) 21.0 ± 1.5 (F)		
Ward et al. (2004), USA [29]	76 F	Mixed sports ^4^, Division I and III NCAA, tertiary	18.8 ± 1.1 (17–21)	59.8 ± 7.1 *	165.6 ± 6.1 *	21.7 ± 2.3		
Wardenaar et al. (2015), Holland [54]	47 (31 M, 16 F)	Mixed sports ^5^, elite, Olympic	21.2 ± 3.9 (18–35)	74.3 ± 10.3	179.3 ± 7.2	21.6 ± 4.1 (17.5–31)		*n* = 1 excluded (incomplete data)

Data are presented as mean ± SD or mean (range) unless otherwise indicated. M: male, F: female, BMI; body mass index, DLW: doubly labeled water study, NR: not reported, BIA: bioelectrical impedance, BW: body mass, EI: energy intake, RMR: resting metabolic rate, UWW: underwater weighing, NCAA: National Collegiate Athletic Association. ^1^ Mixed sports: soccer, tennis, basketball, football, golf, lacrosse, baseball, softball, track and field (T&F), wresting, boxing, ice hockey, skating, dance. ^2^ Mixed sports: endurance sports, ball games or other sports. ^3^ Mixed sports: soccer, basketball, T&F, handball, judo, tennis, volleyball. ^4^ Mixed sports: basketball, cross country, hockey, soccer, volleyball. ^5^ Mixed sports: T&F, cycling, archery, speed skating, short-track skating. ^a^ Athlete calibre and recruitment source not specified. ^ Data for the non-athlete adult and adolescent participants excluded. * Anthropometric data converted to metric units. # Participants assumed >16 years due to recruitment from College sporting clubs or University team.

**Table 2:** Key findings from studies comparing reported energy intake to energy expenditure measured by DLW.

Reference	Dietary Method (Other Methods)	EI (kJ/day)	CHO (%)	Pro (%)	Fat (%)	TEE DLW (kJ/day)	REE (kJ/day)	(TEE–EI)/TEE * 100 (%) (Mean TEE–EI)	Main Findings
Ebine et al. [40]	7-day FR	8900 ± 1700 *				11,500 ± 2800 * (6-day DLW)	5200 ± 300	23.3% * (−2600 ± 3200 kJ)	*r* = −0.854, *p* < 0.05 No change in BW *r* = 0.808, *p* < 0.01 (BW & TEE) *r* = 0.856, *p* < 0.01 (BW & REE) *r* = 0.715, *p* < 0.05 (BW & PAL)
Ebine et al. [21]	7-day FR	13,000 ± 2400 **				14,800 ± 1700 ** (7-day DLW)	7000 ± 300 (eqn.)	12%** (−1800 ± 1200 kJ)	*r* = 0.893, *p* < 0.01 No change in BW
Edwards et al. [41]	7-day FR Food Attitude Scale (30 items) AR	8527 ± 1246 **				12,516 ± 1737 ** (7-day DLW)		32% ** (−3989 ± 2855 kJ)	*r* = −0.83, *p* < 0.01 (i.e., higher TEE, lower reported EI) No change in BW *r* = 0.82, *p* < 0.01 (BW & TEE) (i.e., heavier expended more energy) *r* = −0.74, *p* < 0.01 (BW & EI) (i.e., heavier reported lower EI) *r* = −0.78, *p* < 0.01 (BW & food attitude) (i.e., heavier women reported lower body image scores)
Fudge et al. [42]	7-day FR (W) ActiGraph™ activity monitor	13,241 ± 1330 *	67.3 ± 7.8% (9.8 g/kg)	15.3 ± 4.0% (2.2 g/kg)	17.4 ± 3.9% (1.1 g/kg)	14,611 ± 1043 * (7-day DLW)	6408 ± 222 (eqn.)	13% * (−24 to 9%) (−1370 ± 1738 kJ)	no correlation between EI & TEE *r* = −0.071, *p* < 0.855, No change in BW
Hill and Davies [43]	4-day FR (W)	10,192 ± 2268				12,498 ± 1117 (14-day DLW) *Adj. TEE* 12,983 ± 2268	7150 ± 757	21% (−2791 kJ)	*n* = 8 increased BW (0.3 kg) while reportedly consuming less than real EI. Underreporting of EI not related to % body fat (*r* = 0.11)
Hill and Davies [44]	4-day FR (W)	9263 ± 1309 **				16,556 ± 5100 ** (14-day DLW) *Adj*. *TEE* 14,008 ± 5560	5812 ± 142 (eqn.)	34% ** (−7293 ± 6075 kJ **) *Adj. TEE-E*I −4740 ± 6439 kJ **	*r* = −0.93, *p* < 0.01 Mean TEE adjusted for BW change (−1.2 ± 1.2 kg) *Adjusted r =* −0.93, *p* < 0.01 *Adjusted* (95% LOA—17,619 to 8134 kJ)
Jones and Leitch [22]	10-day test diet (32% fat, 15% pro, 48% CHO) AR	16,297 ± 2598 Adjusted EI 14,410 ± 3870	48%	15%	32%	14,502 ± 4151 (10-day DLW) *Adj. TEE* 14,878 ± 4289		5% (−468 kJ)	No change in BW EI from test diet increased by 10% (*n* = 1); and decreased by 15% (*n* = 1) due to fluctuations in BW and complaint of large portion sizes, respectively. *n* = 1 removed due to disagreement between measures (35%)
Koehler et al. [45]	7-day FR (198 items) 7-day AR (25 items) 24-h N^+^ excretion	14,786 ± 1682		1.38 ± 0.55 g/kg (Ex.) 1.51 ± 0.70 g/kg (Ex. free)		15,196 ± 3598 (7-day DLW)		2.7% (−410 kJ)	Weak association EI & TEE (*r* = 0.48) Removal *n* = 2 (TEE < 1.39 × REE) *Adjusted* correlation (*r* = 0.69, *p* < 0.05) Bland-Altman comparison indicates bias towards underestimating high EI (*p* < 0.01) (95% LOA—5736 to 4912 kJ/day) (−39% to 33% mean EI) No change in BW (*n* = 1 excluded BW change > 3%) Bland-Altman TEE DLW & AR—151 kJ/day (95% LOA—3356 to 3054 kJ/day) *r* = 0.83, *p* < 0.01 (Pro & 24-h urea N^+^) Bland-Altman mean difference Pro & urinary N^−^ 0.01 g/kg/day (95% LOA—0.65 to 0.67 g/kg/day) *r* = 0.95, SEE = 816 kJ/day
Schulz et al. [46]	6-day FR (AR)	9175 ± 1950 (6560–13,359)	59% (333 g/day) (216–612 g/day)	13% (73 g/day) (50–104 g/day)	27% (66 g/day) (49–100 g/day)	11,824 ± 1305 (9832–13,874) (6-day DLW)	7037 ± 351	22% (−925 ± 2301 kJ)	No relationship (*r* = 0.063) No significant change in BW but most lost mass (−84 ± 71 g/day)
Silva et al. [47]	7-day FR #	11,274 ± 2567 *	50.2 ± 3.5% (338.8 ± 82.7 g/day)	18.6 ± 2.6% (125.7 ± 30.5 g/day)	29.4 ± 2.6% (88.1 ± 22.0 g/day)	17,598 ± 3298 * (7-day DLW)	6199 ± 1007 ^	34% (−6837 kJ)	No relationship (*r* = 0.58, *p* = 0.057)
Sjodin et al. [48]	5-day FR (W) (F) 4-day FR (W) (M) (AR)	18,200 ± 1900 (F) (5700–20,200) 30,200 ± 4600 (M) (5400–34,900)	58%	13%	28%	18,300 ± 2200 (F) (7-day DLW) 30,200 ± 4200 (M) (6-day DLW)	5500 ± 300 (F) 7600 ± 300 (M) (eqn.)	1.1 ± 15.7% (F) 0.6 ± 3.3% (M) (−100 ± 1900 kJ)	*r* = 0.96, *p* = 0.0001 No change in BW

EI: energy intake, TEE: total energy expenditure, DLW: doubly labelled water, REE: resting energy expenditure, CHO: carbohydrate, Pro protein, FR: food record, 5-d FR W 5 day weighed food record, 6-d DLW: DLW measured for 6 days, BW: body mass, PAL: physical activity level, eqn. REE: calculated by equation, AR: activity record, LOA: limits of agreement, CI: confidence interval, SD: standard deviation, SEE: standard error of the estimate, N^+^: nitrogen, Ex.: Exercise days, Ex. Free: exercise-free days, Adj.: Adjusted, M: male, F: female. REE: measured by indirect calorimetry unless specified calculated by equation (eqn.). Energy and macronutrient values reported in kcal converted to kJ; macronutrient values reported as percent of energy unless specified in g/kg or g/kg/day. * statistically significant (*p* < 0.05) ** highly significant (*p* < 0.01) # valid dietary records reported for 9 M, 2 F; ^ valid REE reported for 12 M, 6 F, values for CHO, Pro and Fat reported as percent of EI or g/kg/day unless otherwise specified.

**Table 3:** Calculation of weighted mean difference between reported EI and TEE measured by DLW (%).

Reference	*n*	M	F	EI (kJ) (±SD)	TEE (kJ) (±SD)	Difference (%) (TEE–EI kJ)	Weighed Mean Difference (%)
Ebine et al. [40]	9		9	8900 (1700)	11,500 (2800)	22.6 (2600 kJ)	203
Ebine et al. [21]	7	7		13,000 (2400)	14,800 (1700)	12.2 (1800 kJ)	85
Edwards et al. [41]	9		9	8527 (1246)	12,516 (1737)	31.9 (3989 kJ)	287
Fudge et al. [42]	9	9		13,241 (1330)	14,611 (1043)	9.4 (1370 kJ)	84
Hill and Davies [43]	12		12	10,192 (2268)	12,983 (2268)	21.5 (2791 kJ)	238
Hill and Davies [44]	7		7	9263 (1309)	14,008 (5560)	33.9 (4745 kJ)	237
Jones and Leitch [22]	8	5	3	14,410 (3870)	14,878 (4289)	3.1 (469 kJ)	25
Koehler et al. [45]	12	12		14,786 (1682)	15,196 (3598)	2.7 (410 kJ)	32
Schulz et al. [46]	9		9	9176 (1950)	11,824 (1305)	22.4 (2648 kJ)	202
Silva et al. [47]	19	12	7	11,274 (2567)	17,598 (3298)	35.9 (6324 kJ)	683
Sjodin et al. [48]	8	4	4	24,200 (3250)	24,250 (3200)	0.4 (100 kJ)	3
∑ athletes	109	49	60				
			Mean	12,452 kJ (2143)	14,924 kJ (2800)	18% (2477 kJ)	19.1%

**Table 4:** Studies comparing reported dietary intake as measured by two or more methods of dietary assessment.

Reference	Dietary Method	Reference Method(s)	EI (kJ/day)	CHO (g)	Pro (g)	Fat (g)	Main Findings
Baker et al. (2014) [49]	DATA ipad administered modified multiple pass 24-h DR (*n* = 56) (pre-test DATA, *n* = 19)	INTERVIEW 24-h DR (*n* = 56) OBSERVATION RD observed 24-h (*n* = 26)	DATA 14,636 ± 5945 kJ * OBS. 12,728 ± 5280 kJ * DATA 13,870 ± 6117 kJ INTERVIEW 14,041 ± 6627 kJ	DATA 475 ± 190 OBS. 426 ± 159 DATA 449 ± 205 INTERVIEW 449 ± 216	DATA 151 ± 59 OBS. 139 ± 63 DATA 140 ± 62 INTERVIEW 147 ± 77	DATA 116 ± 65 * OBS. 91 ± 53 * DATA 112 ± 73 INTERVIEW 112 ± 61	Significant difference between DATA & OBS. for energy, CHO, Pro, fat, water, sodium, iron, calcium (ICC 0.78–0.91) NS between DATA & INTERVIEW for energy, CHO, Pro or fat (ICC 0.75–0.91) 95% CI between DATA & OBS. NS for CHO 10.1% (−1.2–22.7%) or Pro 14.1% (−3.2–34.5%) but significantly greater for EI * 14.4% (1.2–29.3%) and fat * 26.4% (6.9–49.6%). Additional findings: TEE (DATA + OBS.) 14,836 ± 4012 kJ TEE (DATA + INTERVIEW) 13,117 ± 4305 kJ NS between TEE & EI from DATA, INTERVIEW or OBS. however a tendency for TEE be greater than EI from OBS. method (*p* = 0.104). Good relative validity for DATA for group level comparisons, but large variations of estimates for individual dietary intake, especially athletes with higher intakes (i.e., EI, CHO, Pro).
Braakhuis et al. (2011) [50]	FFQ (70 items)	FR (7d, W) (*n* = 81 FFQ & FR) Biomarker (FRAP) (*n* = 96 FFQ & FRAP) (*n* = 63 FR & FRAP)	14,500 ± 5700 (7500–25,900)	470 ± 190 (53.5 ± 6.8%)	150 ± 80 (17.9 ± 5.8%)	114 ± 54 (28.0 ± 6.2%)	Modest correlation between FR and FFQ antioxidant intake (*r* = 0.38 ± 0.14, 90% CI) Small correlation between biomarker and FFQ (*r* = 0.28 ± 0.15) Additional findings: Correlation highest for antioxidants from cereals (*r* = 0.55 ± 0.11), coffee and tea (*r* = 0.51 ± 0.15); and moderate for vegetables (*r* = 0.34 ± 0.16) and fruit (*r* = 0.31 ± 0.16). FFQ overestimated intake by 42% for those with low intake, and FFQ underestimated by 73% for those with high antioxidant intakes.
Fogelholm & Lahti-Koski (1991) [51]	FUQ (122 items) (FUQ1 participant reported portion size; FUQ2 medium portion sizes)	FR (7d, W)	13,000 ± 2800	397 ± 123	122 ± 31	114 ± 30	Close agreement EI between FR & FUQ1 & FUQ2 EI FR & FUQ1 (95% CI −1.7 to 0.1 ± 4.3) EI FR & FUQ2 (95% CI −0.1 to 1.7 ± 4.0) Additional findings: Mean intake CHO, Pro vit C, calcium, magnesium, iron and zinc overestimated in FUQ1. Mean intake CHO, vit C, calcium, magnesium, iron and zinc from FUQ2 did not differ from FR; however Pro & fat were underestimated. Most food group correlations above *r* = 0.24, except vegetable oils, some other fats, milk, pork, beef and poultry.
Scoffier et al. (2013) ^ [52]	VSSR	FR (1d)	Adolescent athletes, weight sports VSSR 7978 ± 2513 kJ FR 7491 ± 2116 kJ Adolescent athletes, other sports VSSR 7190 ± 2004 kJ FR 7081 ± 1785 kJ				NS between VSSR & FR (adolescent athletes, weight sports) (*p* < 0.11); or between VSSR & FR (adolescent athletes, other sports) (*p* = 0.56).
Sunami et al. (2016) [53]	FFQ (138 food, 20 beverage, 14 seasoning items)	24-h DR (3d, non-consecutive)	24-hDR 13,332 ± 3933 kJ (M) 8962 ± 2117 kJ (F) FFQ 12,159 ± 4996 kJ (M) 8029 ± 2519 kJ (F)	24-hDR 486.6 ± 152.9 (M) 319.9 ± 81.8 (F) FFQ 452.4 ± 182.8 (M) 286.2 ± 76.0 (F)	24-hDR 100.1 ± 32.5 (M) 65.5 ± 16.4 (F) FFQ 82.0 ± 35.5 (M) 59.0 ± 22.8 (F)	24-hDR 83.7 ± 34.2 (M) 64.0 ± 18.6 (F) FFQ 76.7 ± 40.6 (M) 57.1 ± 28.6 (F)	FFQ underestimated EI by 9% M and 10% F Majority nutrients within ± 20% range; largest difference for retinol (77% M, 32% F) Additional findings: For 35 nutrients median deattenuated CC was 0.30 (0.10 to 0.57 (M) and 0.32 (−0.08 to 0.62) (F) For 19 food groups median deattenuated CC was 0.32 (0.17 to 0.72) (M) and 0.34 (−0.11 to 0.58) (F) Lower difference was noted for: cereals, vegetables, fungi food groups; while greater differences noted for: sugar, beverages, seasonings and spices.
Ward et al. (2004) [29]	RAM calcium checklist (54 items)	FR (6d)	Mean calcium (mg/day) RAM 822 ± 331 FR 823 ± 387				Test-retest reliability of RAM was moderate ICC = 0.54 (*p* < 0.0001, *r* = 0.58) RAM had moderate agreement with FR ICC = 0.41 (*p* < 0.0067, *r* = 0.42). RAM correctly identified 84% with low calcium intake based on FR.
Wardenaar et al. (2015) [54]	Compl-eat™ 24-h DR (3d, non-consecutive)	24-h urinary N^+^ excretion; Q (training load, sports foods, dietary supplements)	16,900 ± 4200 (8540–26,600)		24-hDR * 109.6 ± 33 (1.49 ± 0.35 g/kg/day) 24-h N+ excretion * 141.3 ± 38.2 (1.9 ± 0.39 g/kg/day)		Significant mean difference of 25.5 ± 21.3% (−31.7 ± 30 g/day) between 24-hDR and 24-h N^+^ excretion (*p* < 0.001) (*r* = 0.65; 95% CI 0.45–0.79) Additional findings: Underestimation of Pro related to amount of protein intake (*r* = −0.20; 95% CI −0.46 to 0.09) Mean FIL 1.6 ± 0.4 with 78.7% athletes FIL < 1.75 indicating possible under-reporting. Underreporting greater in individuals with higher protein intakes than with lower intakes.

Data are presented as mean ± SD. Energy data (EI, EE) are presented as mean ± SD kJ/day unless otherwise indicated. Energy values reported by authors in Calories converted to kJ. Macronutrient data presented in g/day (or g/kg/day or as percent of energy intake) unless otherwise indicated. ^ Results reported for adolescent athletes only. Data for non-athlete adult and adolescent participants has been excluded. EI: energy intake, TEE: total energy expenditure, CHO: carbohydrate, Pro protein, DATA Digital Dietary Analysis Tool for athletes, 24-h DR: 24 h dietary recall, RD: registered dietitian, ICC: intra-class correlation, CI: confidence interval, NS: non-significant, FFQ: Food Frequency Questionnaire, FRAP: ferric-reducing ability of plasma, FR: food record, FR: (6d, W) Weighed food record measured for 6 days, FUQ: Food Use Questionnaire, VSSR: Virtual Self Service Restaurant, M: male, F: female, RAM: Rapid Assessment Method, N^+^: nitrogen, Q: Questionnaire, FIL: Food Intake Level (FIL = EI/BMR), BMR basal metabolic rate.

**Table 5:** Evaluation of methodological quality of included studies.

References		1. Hypothesis Stated	2. Outcomes Described	3. Subject Characteristics Described	4. Principal Confounders Described	5. Main Findings Described	6. Random Variability provided	7. Actual *p* Value Reported	8. Clinical Significance Reported	9. Biases and Limitations Discussed	10. Representative of Population	11. Participating Subjects Representative	12. Attempt Made to Blind Main Outcome of Intervention	13. Data Dredging Reported	14. Statistical Tests Appropriate	15. Compliance to Intervention Method	16. Measures Used Accurate (Valid And Reliable)	17. Funding and Affiliations Described	18. Recruited over Same Time	19. Adjustment for Confounding	20. Participant Losses Accounted	21. Power
Baker et al. [49]																						
Braakhuis et al. [50]																						
Ebine et al. [40]																						
Ebine et al. [21]																						
Edwards et al. [41]																						
Fogelholm et al. [51]																						
Fudge et al. [42]																						
Hill and Davies [43]																						
Hill and Davies [44]																						
Jones and Leitch [22]																						
Koehler et al. [45]																						
Schulz et al. [46]																						
Scoffier et al. [52]																						
Silva et al. [47]																						
Sjodin et al. [48]																						
Sunami et al. [53]																						
Ward et al. [29]																						
Wardenaar et al. [54]																						
	Yes																					
	No																					
	Unsure/unable to determine																					
	N/A due to DLW methodology																					

## Supplementary Materials

The following are available online at www.mdpi.com/2072-6643/9/12/1313/s1, Supplementary material 1: Search strategy and inclusion criteria; Supplementary material 2: Quality ratings guide.
