# Supplementary material for: Validity of Dietary Assessment in Athletes: A Systematic Review
Source: Nutrients. 2017 Dec 2;9(12):1313. doi: 10.3390/nu9121313 (PMC5748763; doi:10.3390/nu9121313)
Supplement: Supplementary file 1 [file nutrients-09-01313-s001.zip › Supplementary material 1.Search strategy and inclusion criteria.pdf]

Validity of dietary assessment in athletes: A systematic review

Aim: Review of existing literature comparing two or more methods of dietary assessment in athlete populations with dietary intake or diet quality as the outcome measure.

Inclusion criteria	Exclusion criteria
Studies that measure dietary intake in an athletic population to determine validity using two or more methods of nutrition assessment, including dietary biomarkers or reference measures of energy expenditure.<br><br>Studies where the following outcomes are reported but not limited to: energy intake, macro and/or micronutrient intake, food intake, nutritional adequacy, diet quality, or nutritional status.<br><br>Studies in athletes who participate at the collegiate, amateur, professional or elite sport level.<br><br>Studies published 1980-present (ensuring capture of recent developments in technical dietary assessment methodology).	Studies published in a language other than English.<br><br>Studies in children and adolescents (< 16 years of age).<br><br>Pregnant women, general population participants and recreational athletes.<br><br>Conference abstracts and posters.

Search through the following databases: AUSPORT MED (via Informit Online), CINAHL (via EBSCO), EMBASE (via EBSCO), MEDLINE (via OvidSP), SPORTDiscus (via EBSCO) and the Web of Science to extract articles relevant to the topic.

Electronic monitoring established to assess inclusion of eligible recent publications.

Validity of dietary assessment in athletes: A systematic review
Louise Capling[1,6], Kathryn L. Beck[2], Janelle Gifford[1], Gary Slater[3], Victoria M Flood[1,4,5], Helen O'Connor[1,4]

1. The University of Sydney, Discipline of Exercise and Sport Science, Faculty of Health Sciences, Lidcombe, NSW, Australia
2. School of Sport Science and Nutrition, College of Health, Massey University, Auckland, New Zealand
3. School of Health and Sport Sciences, University of the Sunshine Coast, Maroochydore, QLD, Australia
4. The University of Sydney, Charles Perkins Centre, Camperdown, NSW, Australia
5. Western Sydney Local Health District, Westmead, NSW, Australia
6. Queensland Academy of Sport, Sport Performance Innovation and Knowledge Excellence, Brisbane, QLD, Australia

Corresponding author: Louise Capling, The University of Sydney acap7726@uni.sydney.edu.au

Hand search reference lists of selected articles to obtain additional papers for inclusion.

Validity of dietary assessment in athletes: A systematic review
Louise Capling[1,6], Kathryn L. Beck[2], Janelle Gifford[1], Gary Slater[3], Victoria M Flood[1,4,5], Helen O'Connor[1,4]

1. The University of Sydney, Discipline of Exercise and Sport Science, Faculty of Health Sciences, Lidcombe, NSW, Australia
2. School of Sport Science and Nutrition, College of Health, Massey University, Auckland, New Zealand
3. School of Health and Sport Sciences, University of the Sunshine Coast, Maroochydore, QLD, Australia
4. The University of Sydney, Charles Perkins Centre, Camperdown, NSW, Australia
5. Western Sydney Local Health District, Westmead, NSW, Australia
6. Queensland Academy of Sport, Sport Performance Innovation and Knowledge Excellence, Brisbane, QLD, Australia

Corresponding author: Louise Capling, The University of Sydney acap7726@uni.sydney.edu.au

Validity of dietary assessment in athletes: A systematic review

Outline of search terms

Search for DIET

1. Diet*
2. Nutrient*
3. Nutrient adequacy
4. Nutritional adequacy
5. Diet quality
6. Nutritional status
7. Diet variety
8. Food variety
9. Habitual diet
10. Diet* pattern*
11. Eating pattern*
12. Food group*
13. Diet* habit*
14. Nutritional requirement*
15. Sport* nutrition
16. Energy intake
17. 1 or 2 or 3 or 4 or 5 or 6 or 7 or 8 or 9 or 10 or 11 or 12 or 13 or 14 or 15 or 16

Search for NUTRITION ASSESSMENT

18. Diet index
19. Diet quality
20. Diet quality index
21. Diet quality tool*
22. Diet* score
23. Diet ADJ3 score
24. Diet* assessment*
25. Nutrition* assessment*
26. Healthy eating index
27. Diet surveys
28. Nutrition survey*
29. Energy expenditure
30. Energy metabolism
31. Nutritional status
32. Survey*
33. Questionnaire*
34. Screening

Validity of dietary assessment in athletes: A systematic review
Louise Capling[1,6], Kathryn L. Beck[2], Janelle Gifford[1], Gary Slater[3], Victoria M Flood[1,4,5], Helen O'Connor[1,4]

1. The University of Sydney, Discipline of Exercise and Sport Science, Faculty of Health Sciences, Lidcombe, NSW, Australia
2. School of Sport Science and Nutrition, College of Health, Massey University, Auckland, New Zealand
3. School of Health and Sport Sciences, University of the Sunshine Coast, Maroochydore, QLD, Australia
4. The University of Sydney, Charles Perkins Centre, Camperdown, NSW, Australia
5. Western Sydney Local Health District, Westmead, NSW, Australia
6. Queensland Academy of Sport, Sport Performance Innovation and Knowledge Excellence, Brisbane, QLD, Australia

Corresponding author: Louise Capling, The University of Sydney acap7726@uni.sydney.edu.au

35. Checklist
36. Food history or diet history or diet record
37. Food frequency questionnaire
38. 24 hour recall
39. Biological markers
40. Biomarker*
41. 18 or 19 or 20 or 21 or 22 or 23 or 24 or 25 or 26 or 27 or 28 or 29 or 30 or 31 or 32 or 33 or 34 or 35 or 36 or 37 or 38 or 39 or 40

Combine DIET and NUTRITION ASSESSMENT search terms

42. 17 and 41

Search for ATHLETE

43. Athlete*
44. Elite athlete*
45. Collegiate athlete*
46. Competitive athlete*
47. Olympic athlete*
48. International sport*
49. National sport*
50. Team sport*
51. Resistance exercise
52. 43 or 44 or 45 or 46 or 47 or 48 or 49 or 50 or 51

Combine DIET and NUTRITION ASSESSMENT AND ATHLETE search terms

53. 42 and 52

Search for VALIDITY

54. Valid*
55. Compar*
56. Repeatability
57. Reproducibility
58. Reliability
59. Underreporting
60. Under reporting
61. Measurement error*
62. Calibration
63. 54 or 55 or 56 or 57 or 58 or 59 or 60 or 61 or 62

Validity of dietary assessment in athletes: A systematic review
Louise Capling[1,6], Kathryn L. Beck[2], Janelle Gifford[1], Gary Slater[3], Victoria M Flood[1,4,5], Helen O'Connor[1,4]

1. The University of Sydney, Discipline of Exercise and Sport Science, Faculty of Health Sciences, Lidcombe, NSW, Australia
2. School of Sport Science and Nutrition, College of Health, Massey University, Auckland, New Zealand
3. School of Health and Sport Sciences, University of the Sunshine Coast, Maroochydore, QLD, Australia
4. The University of Sydney, Charles Perkins Centre, Camperdown, NSW, Australia
5. Western Sydney Local Health District, Westmead, NSW, Australia
6. Queensland Academy of Sport, Sport Performance Innovation and Knowledge Excellence, Brisbane, QLD, Australia

Corresponding author: Louise Capling, The University of Sydney acap7726@uni.sydney.edu.au

Validity of dietary assessment in athletes: A systematic review

Search for study type and limitations imposed:

Limit 63: English language, human, adult (19 plus years), adolescent, year limit (1980-present).

Validity of dietary assessment in athletes: A systematic review
Louise Capling[1,6], Kathryn L. Beck[2], Janelle Gifford[1], Gary Slater[3], Victoria M Flood[1,4,5], Helen O'Connor[1,4]

1. The University of Sydney, Discipline of Exercise and Sport Science, Faculty of Health Sciences, Lidcombe, NSW, Australia
2. School of Sport Science and Nutrition, College of Health, Massey University, Auckland, New Zealand
3. School of Health and Sport Sciences, University of the Sunshine Coast, Maroochydore, QLD, Australia
4. The University of Sydney, Charles Perkins Centre, Camperdown, NSW, Australia
5. Western Sydney Local Health District, Westmead, NSW, Australia
6. Queensland Academy of Sport, Sport Performance Innovation and Knowledge Excellence, Brisbane, QLD, Australia

Corresponding author: Louise Capling, The University of Sydney acap7726@uni.sydney.edu.au
