# Supplementary material for: Validity of Dietary Assessment in Athletes: A Systematic Review
Source: Nutrients. 2017 Dec 2;9(12):1313. doi: 10.3390/nu9121313 (PMC5748763; doi:10.3390/nu9121313)
Supplement: Supplementary file 1 [file nutrients-09-01313-s001.zip › Supplementary material 2.Quality ratings guide.pdf]

# Assessment of Methodological Quality – Reviewer Guidance

1. *Is the hypothesis/aim/objective of the study clearly described?* (D&B Item 1)

   **Yes** if aim or hypothesis stated; **No** if absent or unclear; **Unsure/Unable to determine** if not obvious and provide comment why.

2. *Are the main outcomes to be measured clearly described in the Introduction or Methods section?* (D&B Item 2)

   **Yes** if outcomes described in Introduction or Method; **No** if first mentioned in Results or not mentioned; or **Unsure/Unable to determine.**

3. *Are the characteristics of the athletes included in the study clearly described?* (D&B Item 3)

   Inclusion/exclusion criteria should be provided.

   **Yes** if included; **No** if not included; or **Unsure/Unable to determine**.

   For example: "*All participants were non-smokers, trained competitively, and all steroid users had been drug-free by self-report at least 6 months prior to the investigation*" would be an acceptable characteristic description. The study must provide sufficient detail e.g. age, type of sport, competitive level, or specify exclusion criteria such as injury, illness, eating disorder.

   Can also consider the following points from the **ADA tool** to assist when reviewing:

   2.1 *Were inclusion/exclusion criteria specified and with sufficient detail and without omitting criteria critical to the study?*

   *2.3 Were health, demographics or other characteristics of the participants described*.

4. *Are the distributions of principal confounders in each group of subjects to be compared clearly described?* (D&B Item 5)

   A list of principal confounders is provided.

   **Yes** if a list of principal confounders is provided; **No** if no confounders provided; or **Unsure/Unable to determine.**

   A confounder is an extraneous variable in a statistical model that correlates (directly or inversely) with both the dependent variable and the independent variable. Confounders in relationships with dietary quality/validation studies may include: dietary supplements, physical activity (unusual or variation from usual pattern).

*Validity of dietary assessment in athletes: A systematic review*

Louise Capling[1,6], Kathryn L. Beck[2], Janelle Gifford[1], Gary Slater[3], Victoria M Flood[1,4,5], Helen O'Connor[1,4]

1. The University of Sydney, Discipline of Exercise and Sport Science, Faculty of Health Sciences, Lidcombe, NSW, Australia
2. School of Sport Science and Nutrition, College of Health, Massey University, Auckland, New Zealand
3. School of Health and Sport Sciences, University of the Sunshine Coast, Maroochydore, QLD, Australia
4. The University of Sydney, Charles Perkins Centre, Camperdown, NSW, Australia
5. Western Sydney Local Health District, Westmead, NSW, Australia
6. Queensland Academy of Sport, Sport Performance Innovation and Knowledge Excellence, Brisbane, QLD, Australia

*Corresponding author*: Louise Capling, The University of Sydney acap7726@uni.sydney.edu.au

# Assessment of Methodological Quality – Reviewer Guidance

Can also consider the following from the **ADA tool:**

3.4 "*If a cohort or cross-sectional study, were groups comparable on important confounding factors*". Potential confounders may be accounted for with the use of exclusion criteria but participants may be excluded from the study if these conditions exist, therefore these would not need to be taken into account in the analyses.

*Validity of dietary assessment in athletes: A systematic review*

Louise Capling[1,6], Kathryn L. Beck[2], Janelle Gifford[1], Gary Slater[3], Victoria M Flood[1,4,5], Helen O'Connor[1,4]

1. The University of Sydney, Discipline of Exercise and Sport Science, Faculty of Health Sciences, Lidcombe, NSW, Australia
2. School of Sport Science and Nutrition, College of Health, Massey University, Auckland, New Zealand
3. School of Health and Sport Sciences, University of the Sunshine Coast, Maroochydore, QLD, Australia
4. The University of Sydney, Charles Perkins Centre, Camperdown, NSW, Australia
5. Western Sydney Local Health District, Westmead, NSW, Australia
6. Queensland Academy of Sport, Sport Performance Innovation and Knowledge Excellence, Brisbane, QLD, Australia

*Corresponding author*: Louise Capling, The University of Sydney acap7726@uni.sydney.edu.au

5. *Are the main findings of the study clearly described?* (D&B Item 6)

   Simple outcome data should be reported for all major findings.

   **Yes** if included; **No** if not included; or **Unsure/Unable to determine**.

6. *Does the study provide estimates of the random variability in the data for main outcomes?* (D&B Item 7)

   In normally distributed data the SE, SD or CIs should be reported. If the distribution of the data is not described, it must be assumed that the estimates used were appropriate and the question should be answered **yes**.

   **Yes** if reported; **No** if not reported; or **Unsure/Unable to determine.**

7. *Have actual probability values been reported for the main outcomes except where the probability value is less than 0.001?* (D&B Item 10)

   **Yes** **No** **Unsure/Unable to determine.**

8. *Was clinical significance as well as statistical significance reported?* (ADA Item 8.6)

   **Yes** if clinical and statistical significance reported; **No** if not reported; or **Unsure/Unable to determine.**

9. *Are conclusions supported by results with biases and limitations taken into consideration?* (ADA Item 9)

   **Yes** study limitations identified **No** **Unsure/Unable to determine.**

   Consider **ADA tool** sub-items when reviewing:

   9.1 Is there a discussion of findings?

   9.2 Are study biases and study limitations identified and discussed?

10. *Were the subjects asked to participate in the study representative of the entire population from which they were recruited?* (D&B Item 11)

    The study must identify the source of participant population and describe how the participants were selected. Athlete participants would be representative if they comprised the entire source population, an unselected sample of consecutive athletes, or a random sample. Random sampling is only feasible where a list of all members of the relevant population exists.

    **Yes** reported; **No** not reported; **Unable to determine** where a study does not report the proportion of the source population from which the athletes are derived.

*Validity of dietary assessment in athletes: A systematic review*


Louise Capling[1,6], Kathryn L. Beck[2], Janelle Gifford[1], Gary Slater[3], Victoria M Flood[1,4,5], Helen O'Connor[1,4]

1. The University of Sydney, Discipline of Exercise and Sport Science, Faculty of Health Sciences, Lidcombe, NSW, Australia
2. School of Sport Science and Nutrition, College of Health, Massey University, Auckland, New Zealand
3. School of Health and Sport Sciences, University of the Sunshine Coast, Maroochydore, QLD, Australia
4. The University of Sydney, Charles Perkins Centre, Camperdown, NSW, Australia
5. Western Sydney Local Health District, Westmead, NSW, Australia
6. Queensland Academy of Sport, Sport Performance Innovation and Knowledge Excellence, Brisbane, QLD, Australia

*Corresponding author*: Louise Capling, The University of Sydney acap7726@uni.sydney.edu.au

# Assessment of Methodological Quality – Reviewer Guidance

*Validity of dietary assessment in athletes: A systematic review*

Louise Capling[1,6], Kathryn L. Beck[2], Janelle Gifford[1], Gary Slater[3], Victoria M Flood[1,4,5], Helen O'Connor[1,4]

1. The University of Sydney, Discipline of Exercise and Sport Science, Faculty of Health Sciences, Lidcombe, NSW, Australia
2. School of Sport Science and Nutrition, College of Health, Massey University, Auckland, New Zealand
3. School of Health and Sport Sciences, University of the Sunshine Coast, Maroochydore, QLD, Australia
4. The University of Sydney, Charles Perkins Centre, Camperdown, NSW, Australia
5. Western Sydney Local Health District, Westmead, NSW, Australia
6. Queensland Academy of Sport, Sport Performance Innovation and Knowledge Excellence, Brisbane, QLD, Australia

*Corresponding author*: Louise Capling, The University of Sydney acap7726@uni.sydney.edu.au

11. *Were those subjects who were prepared to participate in the study representative of the entire population from which they were recruited?* (D&B Item 12)

    The proportion of those asked who agreed should be stated. Validation that the sample was representative would include demonstrating that the distribution of the main confounding factors was the same in the study sample and the source population.

    **Yes** if the proportion of those asked who agreed is stated; **No** if not stated; or **Unsure/Unable to determine**.

12. *Was an attempt made to blind those measuring the main outcomes of the intervention?* (D&B Item 15)

    **Yes** if blinded; **No** if not blinded; or **Unsure/Unable to determine.**

    Can also consider the following from the **ADA tool** as a guide:

    5.2 Were data collectors blinded for outcomes assessment (if outcome is measured using an objective test, such as a lab value, this criterion is assumed to be met)?

13. *If any of the results of the study were based on "data dredging" was this made clear?* (D&B Item 16)

    Any analyses that had not been planned at the outset of the study should be clearly indicated.

    **Yes** if no retrospective unplanned analyses indicated or undertaken; **No** if unplanned analyses were not indicated; or **Unsure/Unable to determine.**

14. *Were the statistical tests used to assess the main outcomes appropriate?* (D&B Item 18)

    The statistical techniques used must be appropriate to the data. For example non-parametric methods should be used for small sample sizes. Where little statistical analysis has been undertaken but where there is no evidence of bias, the question should be answered **yes**. If the distribution of the data (normal or not) is not described it must be assumed that the estimates used were appropriate and the question should be answered **yes**.

    **Yes** **No** **Unsure/Unable to determine.**

    For *validation studies* guidance from **Serra-Majem et al (2009):**

    "*A maximum of 3 points allocated: 1 for comparisons between methods' means, medians or difference; from 0·5 to 1·5 according to the correlation used (crude, energy adjusted, deattenuated or intraclass); plus 0·5 when statistics to assess agreement or misclassification were utilised*."

*Validity of dietary assessment in athletes: A systematic review*

Louise Capling[1,6], Kathryn L. Beck[2], Janelle Gifford[1], Gary Slater[3], Victoria M Flood[1,4,5], Helen O'Connor[1,4]

1. The University of Sydney, Discipline of Exercise and Sport Science, Faculty of Health Sciences, Lidcombe, NSW, Australia
2. School of Sport Science and Nutrition, College of Health, Massey University, Auckland, New Zealand
3. School of Health and Sport Sciences, University of the Sunshine Coast, Maroochydore, QLD, Australia
4. The University of Sydney, Charles Perkins Centre, Camperdown, NSW, Australia
5. Western Sydney Local Health District, Westmead, NSW, Australia
6. Queensland Academy of Sport, Sport Performance Innovation and Knowledge Excellence, Brisbane, QLD, Australia

*Corresponding author*: Louise Capling, The University of Sydney acap7726@uni.sydney.edu.au

# Assessment of Methodological Quality – Reviewer Guidance

Suggest reviewers apply **yes** if appropriate statistics used (i.e. any of the above applied correctly; or other justified and correctly used statistic); or **no** (i.e. none of the above applied, or no justified statistic used or applied incorrectly) rather than rate using a points scale.

*Validity of dietary assessment in athletes: A systematic review*

Louise Capling[1,6], Kathryn L. Beck[2], Janelle Gifford[1], Gary Slater[3], Victoria M Flood[1,4,5], Helen O'Connor[1,4]

1. The University of Sydney, Discipline of Exercise and Sport Science, Faculty of Health Sciences, Lidcombe, NSW, Australia
2. School of Sport Science and Nutrition, College of Health, Massey University, Auckland, New Zealand
3. School of Health and Sport Sciences, University of the Sunshine Coast, Maroochydore, QLD, Australia
4. The University of Sydney, Charles Perkins Centre, Camperdown, NSW, Australia
5. Western Sydney Local Health District, Westmead, NSW, Australia
6. Queensland Academy of Sport, Sport Performance Innovation and Knowledge Excellence, Brisbane, QLD, Australia

*Corresponding author*: Louise Capling, The University of Sydney acap7726@uni.sydney.edu.au

15. *Was compliance with the intervention(s) reliable?* (D&B Item 19)

Where there was non-compliance with the allocated treatment or where there was contamination of one group, the question should be answered **no**. For studies where the effect of any misclassification was likely to bias any association to the null, the question should be answered **yes**.

For example: did the researchers ensure participants were compliant with diet collection methods such as food record? Did the dietitian follow-up on questionable or missing food items? Were there incomplete records (if yes and accounted for in the analysis then the answer is "**yes**", if yes and not accounted for in the analysis then it is "**no**").

**Yes** if compliance was reliable; **No** if not reliable; or **Unsure/Unable to determine**.

16. *Were the main outcome measures used accurate (valid and reliable)?* (D&B Item 20)

For studies where the outcome measures are clearly described, the question should be answered **yes**. For studies which refer to other work or that demonstrates the outcome measures are accurate, the question should be answered as **yes**.

**Yes** **No** **Unsure/Unable to determine.**

Can also refer to the following **ADA items** to assist with the review:

7.2 Were the nutrition measures appropriate to the question and outcomes of concern?

7.3 Was the period of follow-up long enough for important outcomes to occur (such as nutrients of concern)?

7.4 Were the observations and measurements based on standard, valid and reliable data collection instruments and procedures?

7.5 Was the measurement of effect at an appropriate level of precision? (for *validation studies* consider order effect)

7.6 Were other factors accounted for (measured) that could affect outcomes? (i.e. physical activity)

For ***validation studies only***, apply items described by Serra-Majem (2009) to ADA item 7.2-7.6: "*Did the authors consider seasonality, supplements included where appropriate, information gathered by personal interview where necessary*."

17. *Is bias due to study's funding or sponsorship unlikely?* (ADA Item 10)

10.1 were sources of funding and investigator's affiliations described?

10.2 was there no apparent conflict of interest?

*Validity of dietary assessment in athletes: A systematic review*

Louise Capling[1,6], Kathryn L. Beck[2], Janelle Gifford[1], Gary Slater[3], Victoria M Flood[1,4,5], Helen O'Connor[1,4]

1. The University of Sydney, Discipline of Exercise and Sport Science, Faculty of Health Sciences, Lidcombe, NSW, Australia
2. School of Sport Science and Nutrition, College of Health, Massey University, Auckland, New Zealand
3. School of Health and Sport Sciences, University of the Sunshine Coast, Maroochydore, QLD, Australia
4. The University of Sydney, Charles Perkins Centre, Camperdown, NSW, Australia
5. Western Sydney Local Health District, Westmead, NSW, Australia
6. Queensland Academy of Sport, Sport Performance Innovation and Knowledge Excellence, Brisbane, QLD, Australia

*Corresponding author*: Louise Capling, The University of Sydney acap7726@uni.sydney.edu.au

**Yes** if sources of funding, affiliations described or no apparent conflict of interest. **No** if not described or mentioned **Unsure/unable to determine.**

18. *Were study participants in different intervention groups (trials and cohort studies) or were the cases and controls (case-control studies) recruited over the same period of time?* (D&B Item 22)
    **Yes** if recruited over same period of time; **No** if not same time period; or **Unsure/Unable to determine** for a study which does not specify the time period over which athletes were recruited.



Louise Capling[1,6], Kathryn L. Beck[2], Janelle Gifford[1], Gary Slater[3], Victoria M Flood[1,4,5], Helen O'Connor[1,4]

1. The University of Sydney, Discipline of Exercise and Sport Science, Faculty of Health Sciences, Lidcombe, NSW, Australia
2. School of Sport Science and Nutrition, College of Health, Massey University, Auckland, New Zealand
3. School of Health and Sport Sciences, University of the Sunshine Coast, Maroochydore, QLD, Australia
4. The University of Sydney, Charles Perkins Centre, Camperdown, NSW, Australia
5. Western Sydney Local Health District, Westmead, NSW, Australia
6. Queensland Academy of Sport, Sport Performance Innovation and Knowledge Excellence, Brisbane, QLD, Australia

*Corresponding author*: Louise Capling, The University of Sydney acap7726@uni.sydney.edu.au

19. *Was there adequate adjustment for confounding in the analyses from which the main findings were drawn?* (D&B Item 25)

    Answer **no** for trials if the main conclusions of the study were based on analyses of treatment rather than intention to treat; the distribution of known confounders in the different treatment groups was not described; or if the distribution of known confounders differed between the treatment groups but was not taken into account in the analyses.

    In non-randomized studies if the effect of the main confounders was not investigated or confounding was demonstrated but no adjustment was made in the final analyses then the question should be answered as **no**.

    Can also refer to **ADA tool** to assist rating:

    3.4: If confounders are identified, how did the authors manage that? If a cohort or cross-sectional study were the groups comparable on important confounding factors and/or were pre-existing differences accounted for by using appropriate adjustments in statistical analysis?

    **Yes** **No** **Unsure/Unable to determine**.

20. *Were losses of subjects to follow-up taken into account?* (D&B Item 26)

    If the numbers of participants lost to follow-up are not reported, the question should be answered as **unable to determine**. If the proportion lost to follow-up was too small to affect the main findings, the question should be answered **yes**.

    For example: participants who started a study but did not complete it for any reason (e.g. too many other commitments) are considered 'dropouts'. Did the authors acknowledge this?

    **Yes** **No** **Unsure/Unable to determine**.

21. *Did the study have sufficient power to detect a clinically significant effect where the probability value for a difference being due to chance is less than 5%? Sample sizes have been calculated to detect a difference of x% and y%?* (D&B Item 27)

    Did the authors present their reasons for selecting or recruiting the number of participants included in the study? Did they note or discuss the statistical power of the study? If there is mention of the sample size needed to detect a hypothesised difference in the Methods section, or if power is discussed in the Discussion section, or estimates of variance and/or estimates of effect size, then answer **yes**. If there is no report of power or sample size, then answer **no**.

    **Yes** **No** **Unsure/Unable to determine**.

*Validity of dietary assessment in athletes: A systematic review*

Louise Capling[1,6], Kathryn L. Beck[2], Janelle Gifford[1], Gary Slater[3], Victoria M Flood[1,4,5], Helen O'Connor[1,4]

1. The University of Sydney, Discipline of Exercise and Sport Science, Faculty of Health Sciences, Lidcombe, NSW, Australia
2. School of Sport Science and Nutrition, College of Health, Massey University, Auckland, New Zealand
3. School of Health and Sport Sciences, University of the Sunshine Coast, Maroochydore, QLD, Australia
4. The University of Sydney, Charles Perkins Centre, Camperdown, NSW, Australia
5. Western Sydney Local Health District, Westmead, NSW, Australia
6. Queensland Academy of Sport, Sport Performance Innovation and Knowledge Excellence, Brisbane, QLD, Australia

*Corresponding author*: Louise Capling, The University of Sydney acap7726@uni.sydney.edu.au

For *validation studies only* use guidelines provided in Serra-Majem et al. (2009): Apply "**yes**" when sample size is adequate to assess validity (i.e. >100 individuals, or >50 individuals when using biomarker as the gold standard). Suggest apply **yes** if a power calculation has been undertaken.

**References**

Downs, S.H. and Black, N. (1998) The feasibility of creating a checklist for the assessment of the methodological quality both of randomised and non-randomised studies of health care interventions. *Journal of Epidemiology and Community Health* **52**:377-384.

Academy of Nutrition and Dietetics (2016). Evidence analysis manual: steps in the Academy evidence analysis process. Appendix 8: Quality criteria checklist: primary research. Chicago, USA.

Serra-Majem, L., Frost Andersen, L., Henríque-Sánchez, P., Doreste-Alonso, J., Sánchez-Villegas, A., Ortiz-Andrelluchi, A., Negri, E. and La Vecchia, C. (2009) Evaluating the quality of dietary intake studies. *British Journal of Nutrition* **102**:S3-S9.

*Validity of dietary assessment in athletes: A systematic review*

Louise Capling[1,6], Kathryn L. Beck[2], Janelle Gifford[1], Gary Slater[3], Victoria M Flood[1,4,5], Helen O'Connor[1,4]

1. The University of Sydney, Discipline of Exercise and Sport Science, Faculty of Health Sciences, Lidcombe, NSW, Australia
2. School of Sport Science and Nutrition, College of Health, Massey University, Auckland, New Zealand
3. School of Health and Sport Sciences, University of the Sunshine Coast, Maroochydore, QLD, Australia
4. The University of Sydney, Charles Perkins Centre, Camperdown, NSW, Australia
5. Western Sydney Local Health District, Westmead, NSW, Australia
6. Queensland Academy of Sport, Sport Performance Innovation and Knowledge Excellence, Brisbane, QLD, Australia

*Corresponding author*: Louise Capling, The University of Sydney acap7726@uni.sydney.edu.au
